# Quantitative Mapping of Liver Hypoxia in Living Mice Using Time‐Resolved Wide‐Field Phosphorescence Lifetime Imaging

**DOI:** 10.1002/advs.201902929

**Published:** 2020-04-23

**Authors:** Yawei Liu, Yuyang Gu, Wei Yuan, Xiaobo Zhou, Xiaochen Qiu, Mengya Kong, Qingbing Wang, Wei Feng, Fuyou Li

**Affiliations:** ^1^ Department of Chemistry and State Key Laboratory of Molecular Engineering of Polymers Fudan University 220 Handan Road Shanghai 200433 P. R. China; ^2^ Department of Interventional Radiology Ruijin Hospital Shanghai Jiao Tong University School of Medicine 197 Rui Jin Er Road Shanghai 200025 China

**Keywords:** hypoxia, lifetime imaging, liver disease, nanoprobes, quantitative mapping

## Abstract

Hypoxia has been identified to contribute the pathogenesis of a wide range of liver diseases, and therefore, quantitative mapping of liver hypoxia is important for providing critical information in the diagnosis and treatment of hepatic diseases. However, the existing imaging methods are unsuitable to quantitatively assess liver hypoxia due to the need of liver‐specific contrast agents and be easily affected by other imaging factors. Here, a time‐resolved lifetime‐based imaging method is established for quantitative mapping of the distribution of hypoxia in the livers of mice by combining a wide‐field luminescence lifetime imaging system with an oxygen‐sensitive nanoprobe. It is shown that the method is suitable for real‐time quantification of the change of oxygen pressure in the process of hepatic ischemia‐reperfusion of the mouse. Moreover, the developed lifetime imaging methodology is used to quantitatively map liver hypoxia regions in the mouse model of orthotopic liver tumor, where the average oxygen pressure in tumorous liver is far below the normal liver.

## Graphical abstract

A method for quantitative mapping liver hypoxia in living animals based on time‐resolved phosphorescence lifetime imaging is established. The lifetime imaging methodology is successfully applied to quantitatively assess acute liver hypoxia conditions of hepatic ischemia‐reperfusion. Moreover, it is observed that the average oxygen pressure in tumorous liver is much less than in normal liver in the mice bearing orthotopic hepatic tumors.

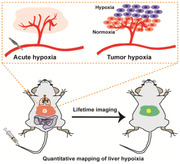

## 1 Introduction

The liver is the main metabolic organ in the human body, and its normal function relies on a sufficient supply of oxygen.^[^ ^1^ ^]^ Distinct changes in oxygen pressure in a regional liver are considered important indicators of hepatocyte dysfunction.^[^ ^2^ ^]^ Liver hypoxia is associated with the pathogenesis of a wide range of hepatic diseases, such as hepatocellular carcinoma (HCC), hepatic ischemia and reperfusion injury, drug‐induced liver injury, liver cirrhosis, liver fibrosis, acute and chronic hepatitis.^[^ ^3^ ^]^ In addition, liver hypoxia is also directly related to liver tumor resistance to radiotherapy and chemotherapy by radiation and chemical antitumor drugs in therapeutic treatment.^[^ ^4^ ^]^ Unfortunately, currently traditional imaging methods are not well suited to quantitatively assess liver hypoxia. The main limitation of these methods is that the used contrast agents are taken up by reticulo‐endothelial system (RES) in normoxic liver as well as in hypoxic liver,^[^ ^5^ ^]^ making it difficult to distinguish the imaging signal of hypoxic region from the whole liver. In view of this situation, only through constructing specific hypoxia tracers with superior metabolism characteristics in the liver could high signal‐to‐noise ratio (SNR) be achieved. In fact, it is difficult to be controllable in liver hypoxia imaging. Therefore, methods for in vivo accurate mapping the distribution of oxygen pressure in hypoxic liver and its surrounding normoxic liver is urgently desired, which not only can provide critical information for the prognosis of various hepatic diseases but also for liver tumor treatment.

Luminescence imaging technique has become a powerful tool for hypoxia imaging in vivo with high sensitivity and high spatiotemporal resolution.^[^ ^6^ ^]^ Currently, most of the reported luminescent in vivo imaging is focused on the intensity‐based imaging method for evaluating hypoxia. However, single luminescent intensity‐based imaging method is vulnerable to influence by the concentration of fluorophores, tissue penetration depth, excitation laser power density, detector gain setting, and so on. Therefore, single luminescence intensity measurements are difficult to perform quantitative detection of liver hypoxia due to above‐mentioned interference factors. Admittedly, ratiometric fluorescent probes can also compensate for many fluctuations from instrumental or environmental factors.^[^ ^7^ ^]^ However, quantitative detection of oxygen concentration in the living animal by constructing ratiometric fluorescent probes still has great challenges. First, it needs to screen different reference fluorophores and emitting fluorophores to construct a reasonable ratiometric system. Second, conventional ratiometric imaging mode has a limited capacity for quantitative detection in vivo due to the highly variable and wavelength‐dependent scattering and absorption process in biological tissue. As another characteristic parameter of luminescence process, lifetime is defined as the average time fluorophores stay in the excited state before returning to the ground state after excitation, resistant to the above influence factors of intensity.^[^ ^8^ ^]^ Recently, two‐photon phosphorescence lifetime microscopy (2PLM) technique has been used to quantitatively measure oxygen pressure in the cerebral vasculature and in the bone marrow,^[^ ^9^ ^]^ but the method based on multiphoton scanning microscope needs ultrafast laser with high excitation power and can only perform multipoint detection of tiny fraction of in vivo.

**Scheme 1:**
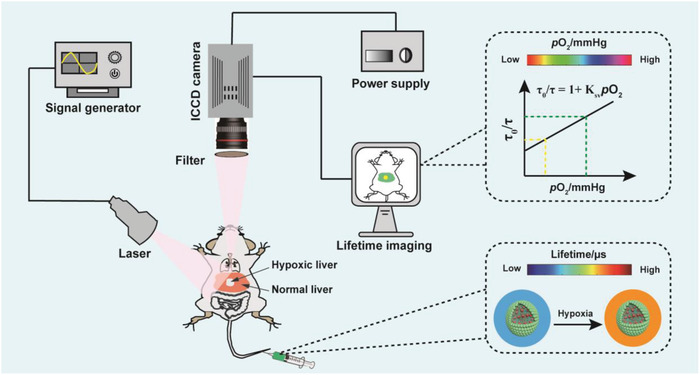
Schematic illustration of phosphorescence lifetime imaging for mapping liver hypoxia in living mice. Wide‐field luminescence lifetime imaging system works as follows: The laser used to excite imaging sample is synchronized by waveform generator to generate a modulated transistor–transistor logic (TTL) pulse. The phosphorescence emission is filtered by optical filter and is collected by time‐gated intensified CCD (ICCD) camera. The images are processed by a computer for data analysis and determination of the lifetime map. Before imaging, an oxygen‐sensitive nanoprobe was administrated by tail vein injection.

In this study, we developed a time‐resolved lifetime‐based imaging method for quantitative mapping the distribution of hypoxia in the livers of mice by combining a wide‐field luminescence lifetime imaging system^[^ ^10^ ^]^ (Scheme S1, Supporting Information) with an oxygen‐sensitive nanoprobe (**Scheme** **1**). We first designed a series of experiments to confirm that luminescence lifetime indeed does not depend on the concentration of fluorophores, tissue penetration depth, excitation laser power density, and detector gain setting in vitro and in vivo. We also demonstrated this method is useful for quantifying monitoring the change of oxygen pressure in acute hypoxia condition, ischemia and reperfusion, in the liver of mouse. Moreover, we observed the wide difference of the oxygen pressure between tumorous liver regions and normal liver regions not only in a subcutaneous tumor model but also in a orthotopic liver tumor model of nude mouse.

## 2 Results

### 2.1 Design, Synthesis, and Characterization of Nanoprobe Pd‐MX

**Figure 1:**
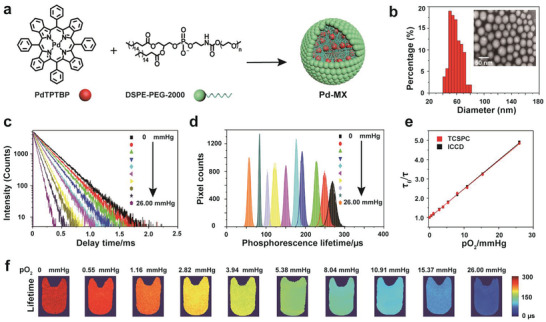
a) Schematic diagram of the synthesis of nanoprobe Pd‐MX. b) TEM image of Pd‐MX (inset) after negative staining with 2% sodium phosphotungstate and particle size distribution in the TEM image. Scale bar, 50 nm. c) Phosphorescence decays of Pd‐MX in aqueous solution at different oxygen levels (0, 0.55, 1.16, 2.82, 3.94, 5.38, 8.04, 10.91, 15.37, and 26.00 mmHg) by time‐correlated single photon counting (TCSPC) technique in the FLS 920 fluorescence spectrometer. d) Phosphorescence lifetime histograms of Pd‐MX in aqueous solution at different oxygen levels (0, 0.55, 1.16, 2.82, 3.94, 5.38, 8.04, 10.91, 15.37, and 26.00 mmHg) by the wide‐field luminescence lifetime measurements systems. e) Stern‐Volmer plots of τ_0_/τ as a function of oxygen pressure, the plots were obtained by time‐correlated single photon counting (TCSPC) technique (red) and wide‐field luminescence lifetime imaging systems (black). f) Phosphorescence lifetime imaging of Pd‐MX in aqueous solution at different oxygen pressure levels (0, 0.55, 1.16, 2.82, 3.94, 5.38, 8.04, 10.91, 15.37, and 26.00 mmHg) by the wide‐field luminescence lifetime measurements systems.

The significant technical progress in measuring oxygen concentration was made by the Wilson and co‐workers, who pioneered the oxygen‐dependent quenching of phosphorescence technique in 1987.^[^ ^11^ ^]^ Afterward, the technique has been revolutionized with a number of advanced sensor chemistries, including Wolfbeis group,^[^ ^12^ ^]^ Papkovsky group,^[^ ^13^ ^]^ Vinogradov group,^[^ ^14^ ^]^ and others.^[^ ^15^ ^]^ These groups have developed numerous phosphorescent probes, measurement methodologies, and instrumentations to perform O_2_ detection by using phosphorescence quenching. In comparison to the methods that indirectly image redox microenvironment,^[^ ^6^, ^16^ ^]^ phosphorescence quenching technique for measuring oxygen^[^ ^17^ ^]^ has several desirable characteristics. First, phosphorescent probes based on transition‐metal complexes can reversibly and directly monitor oxygen. Second, the dependence of phosphorescence lifetime on oxygen pressure follows a well‐defined linear equation, providing an opportunity to quantitatively measure the partial pressure of oxygen (pO_2_). Third, it is a noninvasive optical method and the response time to oxygen is very short. Generally, given aqueous solubility and well‐defined biodistributions, nanoparticle‐based materials are suitable for oxygen measurements in vivo. We designed and synthesized a phosphorescent nanoprobe by the encapsulation of oxygen‐sensitive PdTPTBP into the DSPE‐PEG 2000 phospholipid micelles^[^ ^18^ ^]^ (**Figure** **1**a). The dimension and morphology of the Pd‐MX were characterized by transmission electron microscopy (TEM), which has a near‐spherical shape with a diameter of around 60 nm in its dry state when the Pd‐MX was stained by 2% SPT (sodium phosphotungstate) (Figure 1b). Dynamic light scattering (DLS) measurement shows that the hydrodiameter of Pd‐MX nanoprobes are in a diameter of around 70 nm (Figure S1, Supporting Information), indicating Pd‐MX are monodispersed in aqueous solution. Moreover, Pd‐MX exhibits remarkable stability without significant size change in aqueous solution for 7 d (Figure S2, Supporting Information). Photophysical characterization shows that Pd‐MX has two main absorption maximum peaks at 628 nm and 442 nm, whereas the near‐infrared phosphorescence emission peak is at 794 nm (Figure S3, Supporting Information). There was no aggregation of the Pd‐MX observed in aqueous solutions, as evidenced by the similar absorption spectra of PdTPTBP in the tetrahydrofuran (Figure S4, Supporting Information). In addition, we also tested the data for animal‐like storage stability of Pd‐MX. After incubating Pd‐MX with 50% mouse serum at 37 °C for 48 h, the luminescence intensity basically remain unchanged (Figure S5, Supporting Information), suggesting that nanoprobe Pd‐MX should be stable in vivo.

To examine the optical response of the probe Pd‐MX to oxygen, oxygen quenching experiments based on phosphorescence lifetime measurements from Pd‐MX were performed. The phosphorescence lifetime of Pd‐MX monitored at 800 nm in the aqueous solution decreased nonlinearly with the concentration of increasing oxygen (Figure 1c,d,f). For example, there is a fivefold increase in phosphorescent lifetime of Pd‐MX from 26.00 mmHg oxygen condition to oxygen‐free condition. As illustrated in Figure 1e, a good linear relationship between phosphorescence lifetime (τ_0_/τ) and partial pressure (pO_2_) was observed. To further confirm the standard curve can be used to quantitative mapping of the oxygen pressure of liver and tumor, the Stern–Volmer curves in liver tissue homogenate and in tumor homogenate were measured, which were basically consistent with the curve in aqueous solution (Figure S6, Supporting Information). Meanwhile, the difference of apparent lifetimes between the Stern–Volmer curves obtained by using time‐correlated single photon counting (TCSPC) technique and wide‐field luminescence lifetime imaging systems was found to be minimal (Figure 1e). In addition, we compared the Stern–Volmer calibrations in 28 °C and 37 °C. As shown in Figure S7 (Supporting Information), the Stern–Volmer constant was a slightly increased with rising temperature. However, a temperature change below 9 K does not affect the calibration of the oxygen sensor in 0–15 mmHg oxygen pressure, and can therefore be neglected. Last, we also tested the potential photoinduced O_2_ consumption^[^ ^19^ ^]^ with the liver tissue in living mouse. Even the liver of mouse was irradiated for 6 min by the laser, the luminescence lifetime of the liver in living mouse did not change, indicating that there was no potential light‐induced consumption in the liver of mouse (Figure S8, Supporting Information).

### 2.2 Toxicity Evaluation of Nanomicelles

For potential bioapplication, the cytotoxicity of nanomicelles PdTPTBP/DSPE‐PEG was determined towards HepG2 cells through 3‐(4,5‐dimethylthiazol‐2‐yl)‐2,5‐diphenyltetrazoliumbromide (MTT) assay. Briefly, HepG2 cells were incubated with nanomicelle PdTPTBP/DSPE‐PEG at different concentration (0 × 10^−6^ to 40.0 × 10^−6^ m) in PBS buffer at 37 °C for 24 h. No significant cytotoxicity was observed, and the cell viability was over 85% even with a concentration of 40.0 × 10^−6^ m (Figure S9, Supporting Information). Apart from that, histological analysis experiments were also performed on small animals via hematoxylin and eosin (H&E) stains where the mice were injected intravenously with a Pd‐MX aqueous solution (0.6 mL, 20 × 10^−6^ m). The results showed no obvious histological changes on the organs (heart, liver, lungs, spleen, and kidneys) harvested from nude mice (Figure S10, Supporting Information). Although all these experimental results suggested that Pd‐MX exhibited low toxicity and excellent biocompatibility with living systems, we still inform the users to pay attention to the amount of usage of nanoprobe to avoid minimal toxicity.

### 2.3 Comparison of the Influence Factors of Phosphorescence Intensity and Lifetime

**Figure 2:**
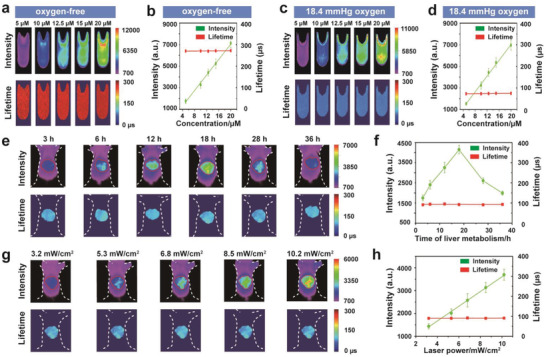
a) Comparison of phosphorescence intensity imaging (top) and lifetime imaging (bottom) of Pd‐MX solutions with different concentrations (5 × 10^−6^, 10 × 10^−6^, 12.5 × 10^−6^, 15 × 10^−6^, and 20 × 10^−6^ m) in the vials under the condition of oxygen‐free. b) The change tendencies of average phosphorescence intensity and average phosphorescence lifetime of Pd‐MX solutions with different concentrations (5 × 10^−6^, 10 × 10^−6^, 12.5 × 10^−6^, 15 × 10^−6^, and 20 × 10^−6^ m) in the vials under the condition of oxygen free. c) Comparison of phosphorescence intensity imaging (top) and lifetime imaging (bottom) of Pd‐MX solutions with different concentrations (5 × 10^−6^, 10 × 10^−6^, 12.5 × 10^−6^, 15 × 10^−6^, and 20 × 10^−6^ m) in the vials under the condition of 18.4 mmHg oxygen pressure. d) The change tendencies of average phosphorescence intensity and average phosphorescence lifetime of Pd‐MX solutions with different concentrations (5 × 10^−6^, 10 × 10^−6^, 12.5 × 10^−6^, 15 × 10^−6^, and 20 × 10^−6^ m) in the vials under the condition of 18.4 mmHg oxygen pressure. e) Comparison of phosphorescence intensity imaging (top) and lifetime imaging (bottom) of the liver in the mouse at different time points after i.v. injection of Pd‐MX solution (0.6 mL, 20 × 10^−6^ m). f) The change tendencies of average phosphorescence intensity and average phosphorescence lifetime of the liver in the mouse at different time points after i.v. injection of Pd‐MX solution (0.6 mL, 20 × 10^−6^ m). g) Comparison of phosphorescence intensity imaging (top) and lifetime imaging (bottom) of the liver under 635 nm laser irradiation with different power density (3.2, 5.3, 6.8, 8.5, and 10.2 mW cm^−2^) after i.v. injection of Pd‐MX solution (0.6 mL, 20 × 10^−6^ m). h) The change tendencies of average phosphorescence intensity and average phosphorescence lifetime of the liver under 635 nm laser irradiation with different power density (3.2, 5.3, 6.8, 8.5, and 10.2 mW cm^−2^) after i.v. injection of Pd‐MX solution (0.6 mL, 20 × 10^−6^ m). Each average intensity values or average lifetime values with error bar values was measured at different positions from the corresponding intensity images or lifetime images (*n* = 5).

Generally, luminescence intensity is susceptible to excitation laser power density, detector gain setting, tissue penetration depth, and fluorophores concentration. We investigated whether luminescence lifetime is affected by these above factors. First, we tested the phosphorescence lifetime change of Pd‐MX solution at different concentrations in the vials. The average phosphorescence lifetime of Pd‐MX (*c* = 5 × 10^−6^ m) was 270 µs in the absence of oxygen. Even though the initial concentration was increased 2×, 2.5×, 3×, and 4× times, the average phosphorescence lifetimes of Pd‐MX were nearly unchanged and the values were 270, 269, 270, and 272 µs, respectively. In contrast, there was an increasing linear relationship between the phosphorescence intensity and the concentration of Pd‐MX (**Figure** **2**a,b). Even in oxygenated samples, for example, 18.4 mmHg, the average phosphorescence lifetimes of Pd‐MX were nearly identical at five different concentrations, which were 73, 73, 74, 75, and 75 µs. As is the case with the oxygen free, the phosphorescence intensity of Pd‐MX increases linearly with the increment of the concentration of Pd‐MX (Figure 2c,d). It is well known that luminescence intensity signals are different when imaging in various excitation laser power. Therefore, the phosphorescence lifetime of Pd‐MX in varying power density of laser was examined. As shown in Figure S11 (Supporting Information), the average phosphorescence intensity increased 2.4‐fold from 2.9 to 7.2 mW cm^−2^, while phosphorescence lifetime of Pd‐MX was almost changeless with the increasing of power density of laser. Similarly, the average phosphorescence intensity jumped along with the increasement of the emICCD gain of camera and the better linear relation existed in the process, however, the average phosphorescence lifetime remained steady (Figure S12, Supporting Information). The influence of phosphorescence lifetime toward tissue penetration depth of light was also evaluated in the vials. The measurement results shown that the phosphorescence intensity signal of Pd‐MX had a five times decrease when the vial was covered with a layer of pork tissue from 0 mm thickness to 5 mm thickness, but the phosphorescence lifetime of Pd‐MX still stabilized in about 272 µs (Figure S13, Supporting Information).

In a further set of experiments, we compared the influence factors of phosphorescence intensity and lifetime in the livers of living mice. The mouse was administered with intravenous (i.v.) injection of Pd‐MX (0.6 mL, 20 × 10^−6^ m) and imaged at different time points after injection. As shown in Figure 2e,f, although the phosphorescence intensity signal climbed up and peaked at about 18 h postinjection and started declining with the concentration change of Pd‐MX in the process of metabolic clearance of the liver, the phosphorescence lifetime in the liver remains about the same. The relationship of phosphorescence lifetime and laser power density in the liver of mouse was also observed (Figure 2g,h). There was a significant increase of the phosphorescence intensity signal in the liver with the phosphorescence lifetime being stable from 3.2 to 10.2 mW cm^−2^. Meanwhile, the phosphorescence lifetime in the liver of mouse was free from influence of the emICCD gain but the phosphorescence intensity in the liver of mouse surges with the increasing emICCD gain (Figure S14, Supporting Information). These results suggested that phosphorescence lifetime is a more robust imaging parameter than the intensity signal for detecting the liver hypoxia.

### 2.4 Lifetime Imaging for Mapping Acute Liver Hypoxia

**Figure 3:**
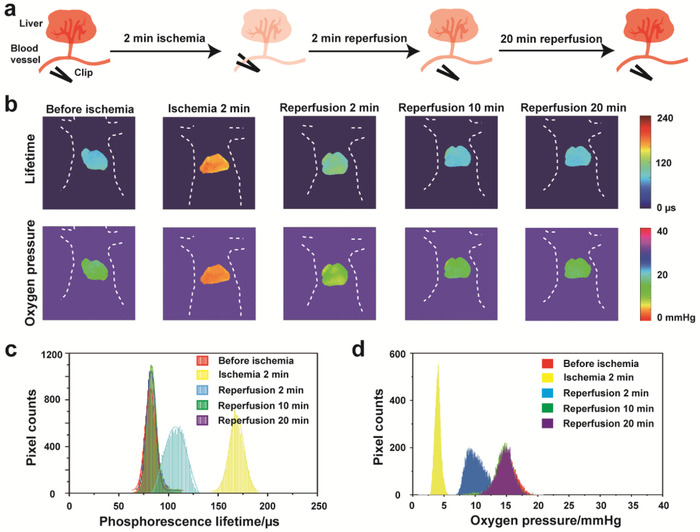
a) Schematic illustration for the hepatic ischemia‐reperfusion model. b) Phosphorescence lifetime imaging of mouse liver recorded during ischemia‐reperfusion (before ischemia, ischemia 2 min, reperfusion 2 min, reperfusion 10 min and reperfusion 20 min). c) Average lifetime histograms of mouse liver obtained before ischemia, ischemia 2 min, reperfusion 2 min, reperfusion 10 min and reperfusion 20 min. d) The oxygen pressure histograms of mouse liver obtained before ischemia, ischemia 2 min, reperfusion 2 min, reperfusion 10 min, and reperfusion 20 min.

Encouraged by the above experiment results, we were curious whether the phosphorescence lifetime imaging method can detect acute hypoxic condition in the liver of mouse. Liver ischemia‐reperfusion (I/R) offers an useful model for the study of acute hypoxia, which is an inevitable procedure particularly following liver transplantation surgery or hepatic resection.^[^ ^20^ ^]^ To demonstrate the feasibility of the imaging method, we first mapped the acute hypoxia condition of liver ischemia‐reperfusion. Liver ischemia was induced in anesthetized mice by cross‐clamping the hepatic artery and portal vein for 2 min, resulting in deprivation of blood flow to approximately of 70% of the liver.^[^ ^21^ ^]^ To induce the process of reperfusion, the clamps were released from the vessels to restore the blood supply (**Figure** **3**a). Before ischemia, the average phosphorescence lifetime of liver in the mouse measured by the luminescence lifetime imaging system was about 84 µs, giving calculated average oxygen pressure of about 15.0 mmHg. We noted that the average phosphorescence lifetime of mouse liver rose sharply to 171 µs after the blood vessels were clamped for 2 min, the corresponding average oxygen pressure of liver dropped down to about 4.0 mmHg. When the clamps were removed to allow the blood flow reperfusion, the average phosphorescence lifetime of mouse liver restored gradually, taking about 2 min to recover to 106 µs (10.6 mmHg). After 10 min of reperfusion, the average phosphorescence lifetime of liver turned back to its original value (before ischemia), and remained at a stable level for 20 min, the average oxygen pressure of liver also returned to 15.0 mmHg (Figure 3b–d). Meanwhile, the variation trend of the phosphorescence intensity during ischemia‐reperfusion was basically consistent with the phosphorescence lifetime. As shown in Figure S15 (Supporting Information), the phosphorescence intensity of mouse's liver increased threefold after an occlusion of blood flow for 2 min by using vascular clamps. During reperfusion, there was a gradual recovery of the phosphorescence intensity of mouse liver after the clamps were released to achieve blood flow reperfusion, and then eventually reduced to a constant level at 10 min. These experimental results clearly demonstrated that the phosphorescence lifetime imaging method can be used to quantitatively monitor the acute hypoxia in the liver of mice.

### 2.5 Lifetime Imaging in the Tumor and Liver of Mouse Bearing Subcutaneous Tumor

**Figure 4:**
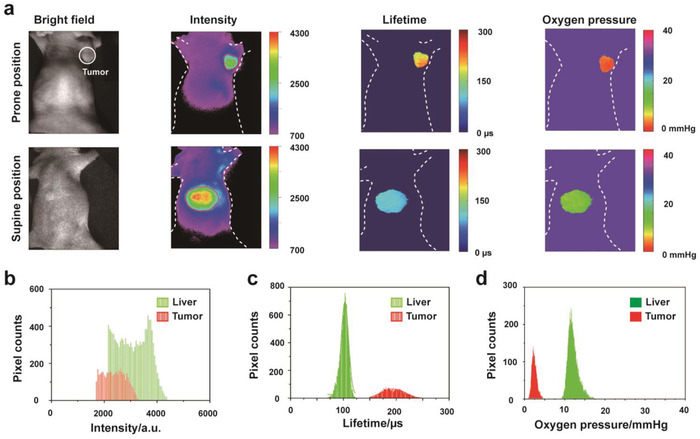
a) Imaging of the living mouse bearing subcutaneously implanted HepG2 tumors after 12 h i.v. injection of Pd‐MX (0.6 mL, 20 × 10^−6^ m). Note: because the tumors were implanted in the back near the right lower hand of mice, so the tumor was performed to image in a prone position and the liver was performed to image in a supine position. b) Phosphorescence intensity histograms of the tumor regions and liver regions in the living mouse bearing subcutaneous tumor. c) Phosphorescence lifetime histograms of the tumor regions and liver regions in the living mouse bearing subcutaneous tumor. d) The oxygen pressure histograms of the tumor regions and liver regions in the living mouse bearing subcutaneous tumor.

To further confirm the potential application of the method for monitoring liver hypoxia, we continuously imaged in the mouse with subcutaneous hepatic tumor. Before imaging, the mouse bearing subcutaneously implanted tumors grown from human liver cancer cell line HepG2 was injected intravenously (tail vein) of Pd‐MX (0.6 mL, 20 × 10^−6^ m). After 12 h postinjection, the intense phosphorescence signal can be found in the tumor of the lateral right lower limb of the mouse. This may be attributed to the small size and the biocompatible polymeric chains of Pd‐MX, facilitating the passive tumor targeting via the enhanced permeability and retention effect (EPR).^[^ ^22^ ^]^ When the mouse was imaged in a supine position, the phosphorescence emission of Pd‐MX can be also seen in the liver of the mouse due to the uptake of reticuloendothelial cells of the liver^[^ ^5b^ ^]^ (**Figure** **4**a). It is noteworthy that phosphorescence intensity signal from the liver organ is stronger than the tumor region (Figure 4b). In contrast, calculating the delay of phosphorescence intensity in the regions of tumor and liver showed that the average phosphorescence lifetime in the liver (98 µs) was less than in the tumor (192 µs) (Figure 4c). Meanwhile, as illustrated in Figure 4d, the average oxygen pressures were confirmed to be in the liver (12.0 mmHg) and in the tumor (2.9 mmHg). Then, the mouse was sacrificed and its organs were resected and further imaged. As illustrated in Figure S16 (Supporting Information), consistent with the results observed in the living mouse, the phosphorescence emission signal can be only seen in the liver and tumor and was insignificant in other organs. The average phosphorescence intensity signal of liver was almost twice as large as the intensity of tumor, whereas the average phosphorescence lifetimes in the isolated liver (188 µs) were still less than in the isolated tumor (249 µs). The calculated oxygen pressures were 3.1 mmHg in isolated liver and 0.7 mmHg in isolated tumor. The reason is that the phosphorescence intensity signal in the tissues is determined by two factors: the concentration of the probe and the level of oxygen pressures. Although the Pd‐MX is accumulated more in the liver of mouse than in the tumor, the oxygen concentration in the normal liver is higher than the tumor tissue, which resulted in the phosphorescent lifetime of the tumor region becoming much longer than the liver tissue. In order to support our speculation, the biodistribution of the Pd‐MX in blood, organs and tissues was further examined at different time points postinjection. As shown in Figure S17 (Supporting Information), the highest accumulation of the Pd‐MX could be found in the liver (16.4–35.5% of injected dose per gram of tissue or ID g^−1^), whereas 6.5–16.5% ID g^−1^ of the Pd‐MX accumulated in the tumor. It should be noted that although we observed that Pd‐MX nanoprobe can enter inside the tumor cells (HepG2) by confocal microscopy in vitro (Figure S18, Supporting Information), it is unclear if the nanoprobe accumulated inside the cell or remained extracellular when nanoprobe Pd‐MX was injected into mice by the tail vein. However, whether inside the cells or outside the cells, it did not have a substantial influence on our measurement results. Meanwhile, immunohistochemistry (IHC) staining for endogenous markers of hypoxia with HIF‐1α antibody also confirmed that the tumor was indeed more hypoxic than the normal liver (Figure S19, Supporting Information), with about 10‐fold higher HIF‐1α staining.

### 2.6 Lifetime Imaging for Mapping Liver Hypoxia in the Mouse Bearing Orthotopic Liver Tumor

**Figure 5:**
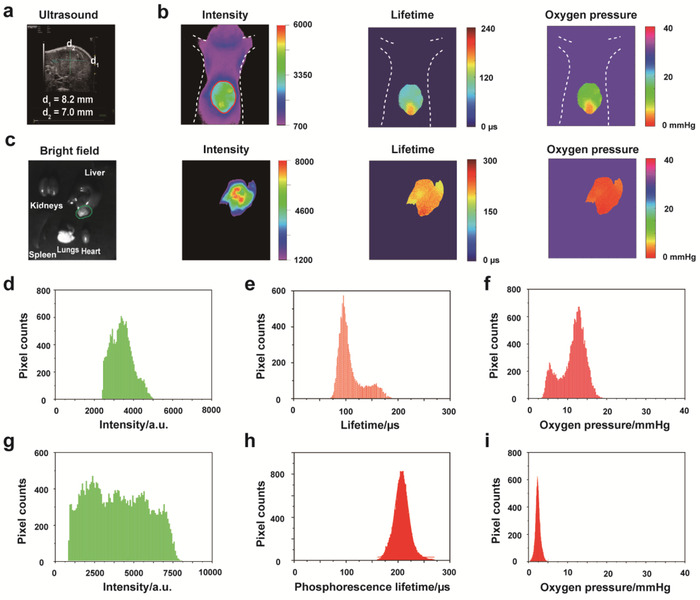
a) Ultrasound imaging of the living mouse bearing orthotopic liver tumor. b) Imaging of the living mouse bearing orthotopic tumor after 12 h i.v. injection of Pd‐MX (0.6 mL, 20 × 10^−6^ m). c) Imaging of isolated organs from the executed mouse bearing orthotopic liver tumor, including heart, spleen, lungs, kidneys, and liver. Green cycle, region of tumorous liver. d) Phosphorescence intensity histograms of the living mouse bearing orthotopic tumor after 12 h i.v. injection of Pd‐MX (0.6 mL, 20 × 10^−6^ m). e) Phosphorescence lifetime histograms of the living mouse bearing orthotopic tumor after 12 h i.v. injection of Pd‐MX (0.6 mL, 20 × 10^−6^ m). f) The oxygen pressure histograms of the living mouse bearing orthotopic tumor after 12 h i.v. injection of Pd‐MX (0.6 mL, 20 × 10^−6^ m). g) Phosphorescence intensity histograms of isolated liver from the executed mouse bearing orthotopic liver tumor. h) Phosphorescence lifetime histograms of isolated liver from the executed mouse bearing orthotopic liver tumor. i) The oxygen pressure histograms of isolated liver from the executed mouse bearing orthotopic liver tumor.

Compared with subcutaneous tumor model, the orthotopic tumor model is closer to clinical applications, and can better reflect the hypoxic tumor microenvironment. To further investigate the potency of the lifetime imaging method in clinical applications, we built an orthotopic model of liver tumor. The mouse was intravenously (i.v.) injected with Pd‐MX (0.6 mL, 20 × 10^−6^ m) and imaged in the luminescence lifetime imaging system at 12 h postinjection. A suspected tumor can be found in the abdomen of the mouse via bright field imaging (Figure S20, Supporting Information). As shown in **Figure** **5**a, ultrasound imaging was further performed in the liver region of suspected tumor and successfully detected a hypoechoic liver tumor with the diameters of 8.2 mm/7.0 mm. Following this, a snapshot of the mouse was taken using the luminescence lifetime imaging system. A strong phosphorescence intensity signal was detected in the liver with very low nonspecific background signals. However, the tumor hypoxia region could not be located using the phosphorescence intensity method because of the low signal to noise ratio between the normal regions and tumor regions of liver (Figure 5d). Interestingly, fitting the delays of phosphorescence intensity of mouse liver, there was a clear difference of phosphorescence lifetime between the normal liver areas and tumor areas. Phosphorescence lifetime histograms of the whole liver regions in the living mouse are also shown clearly two different average lifetimes, 96 and 150 µs (Figure 5e). The oxygen pressure in the normal liver tissue surrounding the tumor was ≈12.4 mmHg, whereas the oxygen in the tumor tissue showed a localized areas of striking hypoxia, areas with average oxygen pressure value about 5.5 mmHg (Figure 5f). To further confirm that the difference of phosphorescence lifetime in the liver of mouse was caused by tumor hypoxia. The mouse was decapitated and sections of heart, liver, spleen, kidneys, and lungs were subjected to further experiments. As shown in Figure 5c, the dissected liver lobe appeared as a medium‐sized milky white tumor. Hematoxylin eosin (H&E) staining also confirmed that the milky white tissue was tumorous in the dissected liver (Figure S21b, Supporting Information). In addition, the phosphorescence intensity imaging for isolated organs indicated that the exclusive signal in the whole‐body mainly came from the liver of mouse (Figure 5c,g). Meanwhile, due to the lack of oxygen supply when the liver organ was culled from the body of mouse, the phosphorescence lifetime of the whole liver tended to increase to 205 µs (Figure 5c,h), the corresponding oxygen pressure of the whole isolated liver was 2.3 mmHg (Figure 5c,i). Last, the remarkably hypoxic microenvironment difference of normal liver regions and tumorous liver region was confirmed by a strong HIF‐1α of Immunostaining (Figure S21c, Supporting Information).

## 3 Discussion

In this study, we established a lifetime‐based imaging method that can quantitatively map the distribution of hypoxia in the livers of mice by combining a wide‐field luminescence lifetime imaging system with an oxygen‐sensitive nanoprobe. In our methodology, the lifetime parameter was chosen instead of the intensity to quantitatively detect liver hypoxia because of the following advantages. First, the luminescence lifetime is unaffected by the factors that influence intensity signals, such as laser power, tissue penetration depth, and detector gains. Second, the contrast agents including PET imaging and luminescent intensity imaging are inevitably taken up by hypoxic liver as well as normoxic liver when the contrast agents are injected by the intravenous administration, making it difficult to obtain controllable information of contrast agent concentrations between the hypoxic liver and normoxic liver. On the contrary, lifetime does not change with the difference of contrast agent concentrations. So even there is only a slight difference of contrast agent concentrations between normoxic and hypoxic liver regions, detecting liver hypoxia can be achieved by using lifetime imaging method. Our experimental results are consistent with the above theory, in which phosphorescence lifetime is indeed independent of fluorophore concentration, tissues penetration depth, laser power density, and detector gains in vitro and in vivo.

The decision to use wide‐field luminescence lifetime imaging to quantitatively detecting liver hypoxia in living animals, rather than two‐photon phosphorescence lifetime imaging microscopy (PLIM) technology,^[^ ^9^ ^]^ frequency domain PLIM,^[^ ^23^ ^]^ or wide‐field TCSPC imaging,^[^ ^24a^ ^]^ was based both on practical and hypothetical considerations. This technique based on two‐photon microscope needs ultrafast laser with high excitation power and can only perform multipoint detection of tiny fraction of living tissue. Besides, two‐photon PLIM often shows slow image frame rate of minutes or even hours for long dye lifetime (τ > 1 µs), since the phosphorescence delay signals for each pixel need to be sequentially collected and analyzed. In fact, the slow imaging rate makes it inaccurate and inconvenient when measuring the oxygen pressure of tissues in living mice because it is hard to keep the mouse in the same position for a long time. The frequency domain method is actually a good alternative in our imaging setup, but has basically the same timing resolution and acquisition time. Wide‐field TCSPC imaging method has the unique characteristics of single photosensitivity and wide‐field data collection. However, the time resolution of the method is limited by the frame rate of the camera. Recently, the next generation camera, TimepixCam improves time resolution to nanosecond.^[^ ^24b,c^ ^]^ The photo counting wide‐field lifetime imaging with TimepixCam may show enticing prospect for oxygen sensing in the future. Compared with the 2PLIM, our self‐built luminescence lifetime imaging system is based on wide‐field time‐gated detection technology. The lifetime imaging system based on time‐gated data acquisition consists of an excitation light source, a photodetector (ICCD camera that can provide <500 ps gate width), a delay device, and an image processing device. By using the wide‐field luminescence lifetime imaging system, faster system imaging speed can be achieved because the imaging signals from all pixels are collected at once. Moreover, the imaging system can provide cm‐sized fields of view, which is well suited for imaging of liver, spleen, gastric, bladder, or lung. Due to the intrinsic nature of the unfocused beam, this system lacks 3D spatial information, but other 3D reconstruction methods, e.g., optical tomography, may be integrated to produce more spatial information. In addition, the imaging system enables the direct measurement of sample lifetime between tens of microseconds and milliseconds, but cannot measure the sample lifetime in the nanosecond region because of the laser falling edge (10 µs). If measuring several microseconds or nanoseconds luminescence lifetimes, we need to use expensive nanosecond or picosecond pulse lasers and waveform generator with larger bandwidth.

In summary, a new method for quantitatively detecting liver hypoxia condition in the living mice has been described. We first evaluated the change of oxygen pressure of acute liver hypoxia in the mouse model of liver ischemia‐reperfusion by using the imaging technique. The changes of oxygen pressure can be clearly seen in the process of liver ischemia‐reperfusion, in which the oxygen pressure sharply decreased after ischemia 2 min and returned to the level of pre‐ischemia after 10 min reperfusion (Table S1, Supporting Information). Also, we observed different oxygen pressure between the tumor and liver regions of the mouse bearing subcutaneous tumor using lifetime imaging method, but the phosphorescence intensity imaging method shown opposite result in the two regions. In addition, in order to bring the technology closer to clinical application, we built the mouse model bearing orthotopic liver tumor, where a clear hypoxic map has been drawn in the whole liver of the mouse by the imaging method. In contrast, quantitative mapping of liver hypoxia in the mouse model bearing orthotopic liver tumor is difficult to achieve by intensity‐based imaging method. It is worth noting that the average oxygen pressures of normal liver in the mice measured by the wide‐field lifetime imaging method were 10.9–15.0 mmHg (Table S1, Supporting Information), which are in close agreement with results measured by oxygen electrode in the reported literatures (17.5 ± 2 mmHg in rats;^[^ ^25^ ^]^ 17 ± 4 mmHg in rats;^[^ ^26^ ^]^ median 23 mmHg in rats^[^ ^27^ ^]^). We also found subcutaneous tumors of different sizes had different oxygen pressures (2.9, 6.3, and 2.4 mmHg) (Figure S22 and Table S2, Supporting Information). The measured oxygen pressures of the tumorous livers in the mice bearing orthotopic liver tumors were substantially below those of normal liver tissues (Table S3, Supporting Information). Last, we expect that this wide‐field time‐resolved imaging method for quantitative mapping of liver hypoxia can provide an opportunity for the diagnosis and treatment of hepatic hypoxic diseases.

## 4 Outlook

Our developed method for liver hypoxia imaging of living small animal combines the advantages of metal porphyrin with wide‐field lifetime imaging. Nanomicelle probe based on near‐infrared Pd‐porphyrin dye is ideally suitable for oxygen measurement in tissue because of their excellent analytical characteristics such as high oxygen sensitivity, good biological compatibility, NIR emission. Moreover, wide‐field phosphorescence lifetime imaging can perform faster imaging rate than traditional PLIM, where scanning a picture needs to take long acquisition time. The approach is beneficial especially in life science application where it allows long‐term monitoring the change of oxygen pressure of living tissues or organisms. With the development of more sensitive oxygen probes and further optimization of imaging devices and equipments, we believe the combination of suitable probes with wide‐field phosphorescence lifetime imaging will facilitate further studies on preclinical whole‐body hypoxia imaging.

## 5 Experimental Section

##### Synthesis of Nanoprobe Pd‐MX

Typically, 1.84 mg PdTPTBP was dissolved in 1 mL tetrahydrofuran (THF) to prepare phosphorescent dye stock solution (2 × 10^−3^ m) and 10 mg 1, 2‐distearoyl‐sn‐glycero‐3‐phosphoethanolamine–poly(ethylene glycol) (DSPE‐PEG, *M* = 2000) was dissolved in 2 mL THF to prepare DSPE‐PEG‐2000 solution. Then 20 µL of the dye stock solution was added to 2 mL of above phospholipid PEG 2000 solution and was mixed by ultrasonic concussion. The mixture solution was pipetted and quickly added into the 2 mL stirred pure water. After stirring for 5 min in room temperature, the mixture was evaporated the organic solvent by a rotary evaporator and filtered through aqueous phase filter with 0.13 µm pore size. The nanomicelle solution was stored in the 4 °C refrigerator for the next use. There is no observable precipitation, aggregation, photobleaching and dissociation even for one month of storage.

##### Cell Culture and Cell Cytotoxicity of Pd‐MX

HepG2 cells from a human liver cancer cell line were obtained from the Institute of Biochemistry and Cell Biology, Shanghai Institutes for Biological Sciences (SIBS), Chinese Academy of Sciences (CAS, China). HepG2 cells were grown at 37 °C and 5% CO_2_ by the supplement to use the culture medium of Eagle's medium (MEM) blended with 10% fetal calf serum (FBS). The cell cytotoxicity of Pd‐MX was performed by using methyl thiazolyl tetrazolium (MTT) assay on HepG2 cells. In brief, HepG2 cells were plated in 96‐well plates and incubated at the above same temperature and CO_2_ concentration in total for 24 h. The HepG2 cells were then added with various concentrations of new synthetic nanomicelle probes (0 × 10^−6^, 1.0 × 10^−6^, 2.0 × 10^−6^, 3.0 × 10^−6^, 4.0 × 10^−6^, 5.0 × 10^−6^, 10.0 × 10^−6^, 20.0 × 10^−6^, 40.0 × 10^−6^ m) under the dark environment. After further incubation of 24 h, MTT (20 µL, 5 mg mL^−1^) was respectively treated to every well and the HepG2 cells were grown at the above same temperature and CO_2_ concentration for another 4 h. The generating purple formazan products were dissolved in dimethyl sulfoxide (DMSO) and the absorbance of products at 490 nm was measured in a Bio Tek ELX 800 absorbance microplate readers. The viability of cell was calculated using the following formula: cell viability (%) = (absorbance values of treatment group/absorbance value of control group) x 100%.

##### Luminescence Lifetime Imaging System

Luminescence lifetime imaging system is designed according to the following principles. As shown in Scheme S1 (Supporting Information), a waveform generator (UTG2025A, Uni‐Trend Co. Ltd.) is connected to a fiber‐coupled laser (MW‐RL‐635/1‐1300 mW, Changchun Laser Optoelectronics Technology Co. Ltd.) to generate a modulated transistor–transistor logic (TTL) signal, and then the laser is used to excite the sample on the sample stage. It is noted that the described imaging setup can be modified by LED if LED can be modulated to produce short and well‐defined pulses. The falling edge of the LED should be short enough (typically below 10 µs for measuring lifetime of more than 30 ms) to meet the need for time‐gated detection. The emission beam is cleaned by a band pass filter (FF01‐800/12, Semrock) loaded on the camera lens before being collected by the emICCD camera with an InGaAs enhancer (PI‐MAX4‐1024B/EM, <500 ps gate width, 1024 × 1024 pixels, Princeton Instruments). A series of phosphorescence delay images are collected by controlling the delay time of the time gate. Briefly, the laser is operated at 1000 Hz and 20% duty cycle by the wavefrom generator, the first time gate of 20 µs is used to avoid the falling edge of the laser, the images of 20–720 µs delay (a total of 29) are collected by the emICCD camera with an InGaAs enhancer (gate width 50 µs, step length 50 µs). The phosphorescence intensity at time *t* was be expressed by the following equation

(1) $$I_{t}=I_{0}\times e^{-t/\tau }+C$$

where *I_t_* and *I*_0_ are, respectively, the phosphorescence intensity in the delay time, *t*, and before the delay, *t* is the delay time of the time gate, *C* is the background signal of the camera. The phosphorescence lifetime is fitted by the equation given above by the phosphorescence intensity signal extracted from the time‐gate images. It is notable that the scattered signals and the background signals have also the same delay curve and are mistakenly considered to be phosphorescence. Therefore, the image area indicated by the lifetime is extracted from the phosphorescence image by the threshold method. All calculations are completed using MATLAB image processing software.

##### Lifetime Imaging for Mapping Acute Liver Hypoxia

The aqueous solution of the Pd‐MX probe (0.6 mL, 20 × 10^−6^ m) was intravenously injected into the node mice (6 weeks of age) through the tail. After 12 h of administration, the mice were anesthetized by the isoflurane whose limbs were fixed in a supine position. The abdomen of mouse was disinfected with 1% iodine and was cut with a tissue scissors layer by layer to expose the abdominal cavity. Vascular of the middle lobe and left lobe of the liver and bile ducts were clamped with noninvasive vascular clamps, resulting in 70% ischemia of the liver while retaining blood supply from the right and caudate lobe of the liver. After blocking, the color of the liver in the blocked area changed from bright red to dark red which indicates that the ischemia liver model was successfully built. The vascular clamps were removed to construct the liver reperfusion model after ischemia 2 min. Before and after ischemia‐reperfusion, the mice were imaged using the luminescence lifetime imaging system.

##### Lifetime Imaging in the Mice Bearing Subcutaneous Tumor

Mice handing was approved by the guidelines of the Institutional Animal Care and Use Committee, school of Pharmacy in Fudan University. To construct a hepatocellular carcinoma HepG2 tumor model subcutaneously implanted in mice, logarithmic growth stage HepG2 cells (1 × 10^7^/0.2 mL) were subcutaneously injected into the lateral right lower limb of the mice (4 weeks of age). After about 20 d, the tumor grew to a diameter of about 5–10 mm and can be used in subsequent imaging experiments. The aqueous solution of the Pd‐MX probe (0.6 mL, 20 × 10^−6^ m) was injected into the tumor‐bearing mice through the tail vein. At various time points after the injection, the mice were anesthetized with isoflurane gas, and then placed under wide field luminescence lifetime measurements system for imaging. After the 12 h imaging time point, the mice were killed, and the tumor and other major organs were dissected and immediately imaged under the living lifetime imaging system.

##### Lifetime Imaging in the Mice Bearing Orthotopic Liver Tumor

The mouse model of orthotopic liver tumor was built according the following main operational steps. Taking a well‐growing subcutaneous tumor, and cutting it into a tumor tissue block of about 2 mm × 2 mm × 1 mm by using the ophthalmology. The mice (6 weeks of age) were subjected to gas anesthesia using a gas anesthesia system, and the anesthetic was isoflurane. After the animal was anesthetized, the supine position was taken, and the skin was cut with a tissue scissors layer by layer. The opening length was about 1 cm, and the liver was exposed. Then a trocar was used to gently poke a tunnel about 3 mm deep in the middle of the lobe of the liver. In the process, sterile cotton swabs were used to gently press the blood to stop the blood. The prepared tumor tissue block was carefully placed into the tunnel, and then bioadhesive (OB glue) was chosen to bond the wound before closing the abdomen. After about 3 weeks, the tumor grew to a diameter of about 5–10 mm and can be used in subsequent imaging experiments. The aqueous solution of the Pd‐MX probe (0.6 mL, 20 × 10^−6^ m) was injected into the tumor‐bearing mice through the tail vein. After the injection, the mice were imaged as well as the above subcutaneous tumor mice.

## Conflict of Interest

The authors declare no conflict of interest.

## Supporting information

Supporting Information
