# Supplementary material for: Quantitative Mapping of Liver Hypoxia in Living Mice Using Time‐Resolved Wide‐Field Phosphorescence Lifetime Imaging
Source: Adv Sci (Weinh). 2020 Apr 23;7(11):1902929. doi: 10.1002/advs.201902929 (PMC7284196; doi:10.1002/advs.201902929)
Supplement: Supplementary file 1 — Supporting Information [file ADVS-7-1902929-s001.pdf]

Copyright WILEY-VCH Verlag GmbH & Co. KGaA, 69469 Weinheim, Germany, 2020.

ADVANCED SCIENCE
Open Access

Supporting Information

for *Adv. Sci.,* DOI: 10.1002/advs.201902929

Quantitative Mapping of Liver Hypoxia in Living Mice Using Time-Resolved Wide-Field Phosphorescence Lifetime Imaging

*Yawei Liu, Yuyang Gu, Wei Yuan, Xiaobo Zhou, Xiaochen Qiu, Mengya Kong, Qingbing Wang, Wei Feng, and Fuyou Li**

Supporting Information

Quantitative Mapping of Liver Hypoxia in Living Mice Using Time-Resolved Wide-Field Phosphorescence Lifetime Imaging

Yawei Liu[1#], Yuyang Gu[1#], Wei Yuan[1], Xiaobo Zhou[1], Xiaochen Qiu[1], Mengya Kong[1], Qingbing Wang[2], Wei Feng[1] and Fuyou Li[1]*

Dr. Y. Liu, Dr. Y. Gu, Dr. W. Yuan, Dr. X. Zhou, Dr. X. Qiu, Dr. M. Kong, Prof. Dr. W. Feng, Prof. Dr. F. Li

Department of Chemistry and State Key Laboratory of Molecular Engineering of Polymers, Fudan University, 220 Handan Road, Shanghai 200433, P. R. China

Dr. Q. W

Department of Interventional Radiology, Ruijin Hospital, Shanghai Jiao Tong University School of Medicine, 197, Rui Jin Er Road, Shanghai, 200025, China.

[#]These authors contributed equally.

E-mail: fyli@fudan.edu.cn

General methods

The used chemicals without any further purification were acquired from Sigma-Aldrich chemical reagent Co. Ltd.. All reactions that are sensitive to air were carried out under the protection of Ar atmosphere. The compounds were characterized by ^{1}H NMR, ^{13}C NMR, or (and) MALDI-Tof/Tof mass spectra (MALDI-Tof/Tof-MS). ^{1}H and ^{13}C NMR spectra were measured by using a Bruker Ultra Shield Plus 400 MHz NMR Instruments at 25 °C. Mass spectrometry is measured using a AB SCIEX 5800 MALDI-TOF/TOF mass spectrometer. The UV-visible absorption spectra were obtained with Shimadzu UV-2550 UV−vis-NIR spectrometer. Photoluminescence spectra were measured using an an Edinburgh LFS-920 fluorescence spectrometer with 450 W xenon lamp. The size and morphologies of the nanoparticles were characterized through a transmission electron microscope (JEOL, JEM-2010F) at a working voltage of 200 kV. DLS and zeta potential were carried out on a Malvern Zetasizer Nano ZS system. For oxygen sensing, two mass flow controller (HORIBA, S600, 5 SCCM and 10 SCCM) were used for controling O_2 concentration, the mass flow controller of 5 SCCM was linked to mixing gas cylinder (O_2/Ar), another one of 10 SCCM was linked to pure Ar gas cylinder. The different concentrations of oxygen were controlled by mixing different proportions of gas, before which the oxygen contents in the cylinders were corrected by the oxygen detector. Biodistribution of Pd-MX in different organs at various time points were detected for Pd element by ICP-MS.

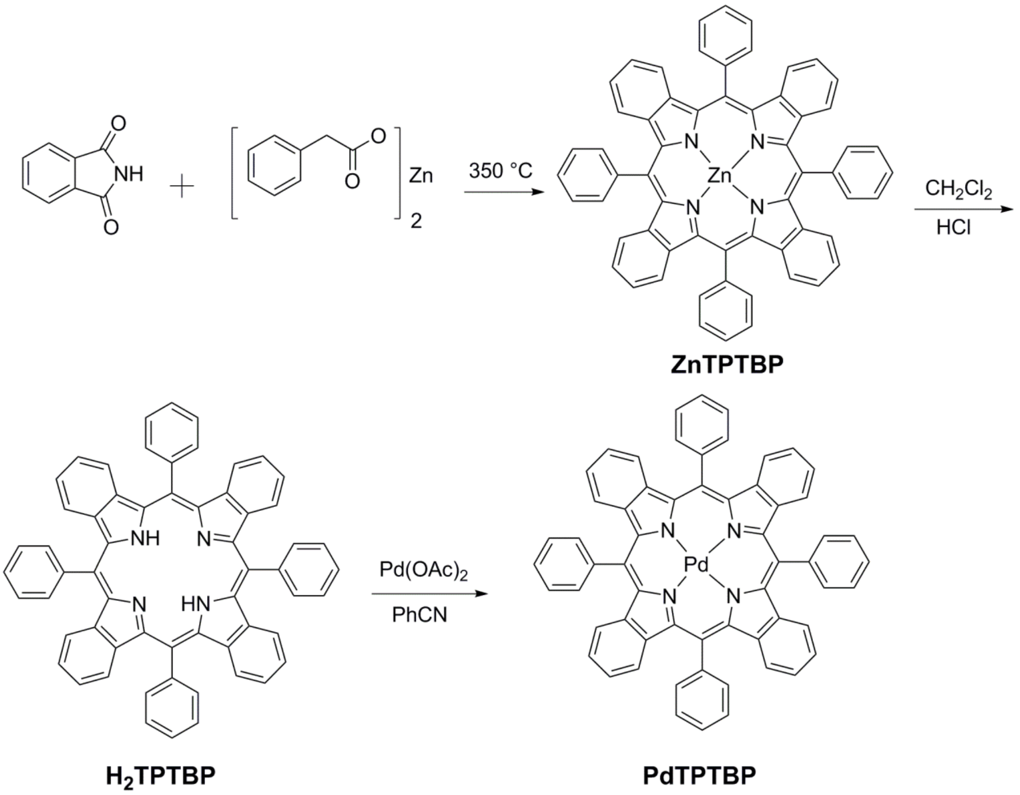

Compound ZnTPTBP, H2TPTBP and PdTPTBP were synthesized according to the reported procedures[1,2].

Syntheis of ZnTPTBP. Zinc phenylacetate (4.98 g, 14.9 mmol) and phthalimide (5.41 g, 36.8 mmol) were added into the mortar to evenly mix. After drying at 105 °C, the reaction was carried out in the closed stainless steel reaction kettle and was heated at 350 °C for 1.5 h in a muffle furnace. The melt was cooled and washed with hot water (2×100 mL) and was dissolved in CH_2Cl_2. The organic layer was evaporated to dryness and purified on silica gelcolumn chromatography. Yield: 1.46%, 320 mg. ^{1}H NMR (400 MHz, $CDCl_3$): δ 8.31 (d, J = 6.8 Hz, 8H), 7.99 (t, J = 7.2 Hz, 4H), 7.60 (d, J = 7.2 Hz, 8H), 7.25 (d, J = 4.8 Hz, 8H), 7.16 (d, J = 4.0 Hz, 8H). Maldi-Tof/Tof-MS: calcd. for $C_{60}H_{36}N_4Zn^+$ 876.2231 $[M]^+$, found 876.1817 $[M]^+$.

Syntheis of H_2TPTBP. ZnTPTBP (43.8 mg, 0.05 mmol) was added the mixture solvents of 10 mL CH_2Cl_2 and 10 mL hydrochloric acid solution ($HCl:H_2O$ = 1:2). After stirring for 0.5 h, water (30 mL) was added in the mixture. The organic layer was evaporated to dryness and

purified on silica gelcolumn chromatography.^{1}H NMR (400 MHz, CD_2Cl_2):δ 8.40 (d, J = 6.4 Hz, 8H), 8.01 (t, J = 7.6 Hz, 4H), 7.96 (d, J = 7.6 Hz, 8H), 7.33 (m, 16H). Maldi-Tof/Tof-MS: calcd. for $C_{60}H_{38}N_4^+$ 814.3096 $[M]^+$, found 814.2847 $[M]^+$.

Synthesis of PdTPTBP. The compound of PdTPTBP was synthesized by the coordination of H_2TPTBP and palladium acetate at high temperature. Typically, under the protection ofN_2atmosphere, H_2TPTBP (36 mg, 0.045 mmol) was dissolved in 5 mL of benzonitrile solution. Then palladium acetate (30 mg, 0.13 mmol) was added to the reaction mixture and refluxed at 180 ° C. The weak absorption peak at 633 nm in the toluene solution faded away, and a new strong absorption peak appeared at 628 nm by the monitoring of UV-Vis absorption spectrum. After the reaction was completed, the mixture was cooled to room temperature and then purified by silica gel chromatography. Yield: 23 mg (56.4%). ^{1}H NMR (400 MHz CD_2Cl_2): δ 8.30 (d, J = 7.2 Hz, 8H), δ 8.03 (t, J = 7.2 Hz, 4H), 7.94 (d, J = 7.6 Hz, 8H), 7.21 (m, 16H). Maldi-Tof/Tof-MS: calcd. for $C_{60}H_{36}N_4Pd^+$ 918.1975 $[M]^+$, found 918.1699 $[M]^+$.

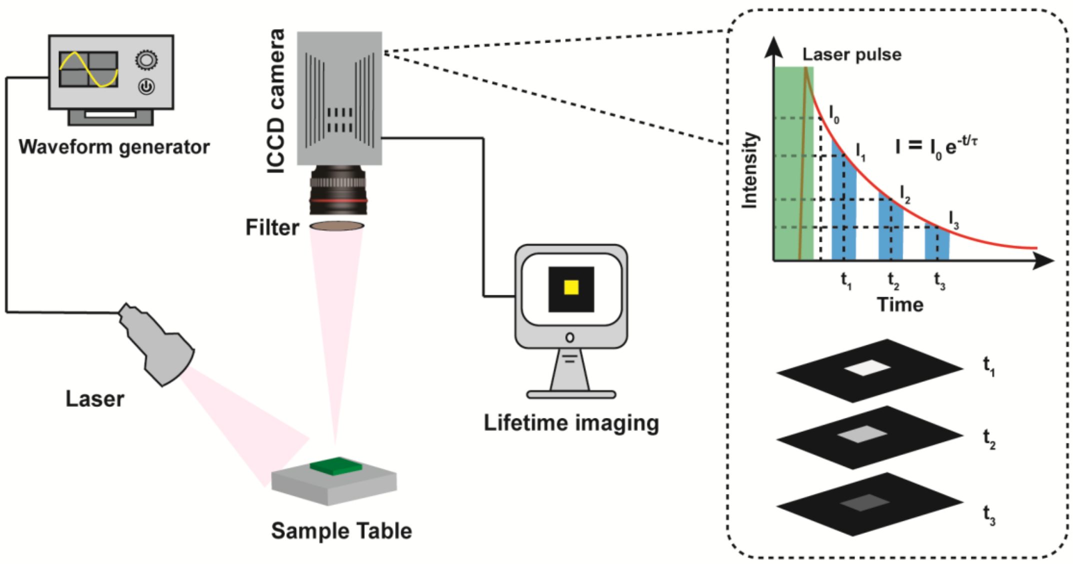

Scheme S1. The schematic illustration of time-resolved imaging system. The laser is controlled by waveform generator to generate a modulated transistor–transistor logic (TTL) signal, and then is used to excite imaging sample. The emission beam is cleaned by a band pass filter loaded on the camera len before being collected by the emICCD camera with an InGaAs intensifer. After collecting all the images, the images are further processed by a computer for data analysis and determination of the lifetime map (wrote in MATLAB code).

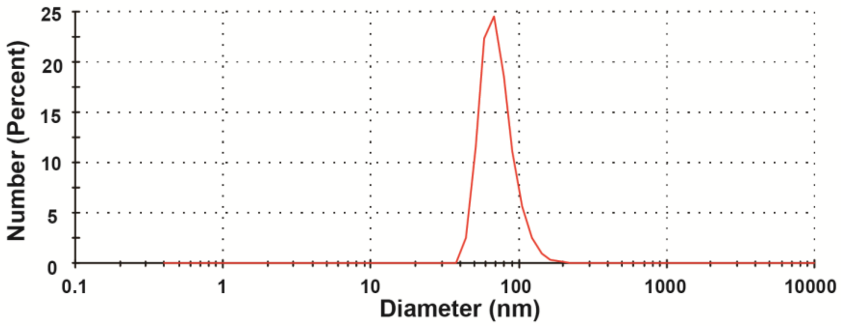

Figure S1. DLS analysis of number size of Pd-MX in distilled water.

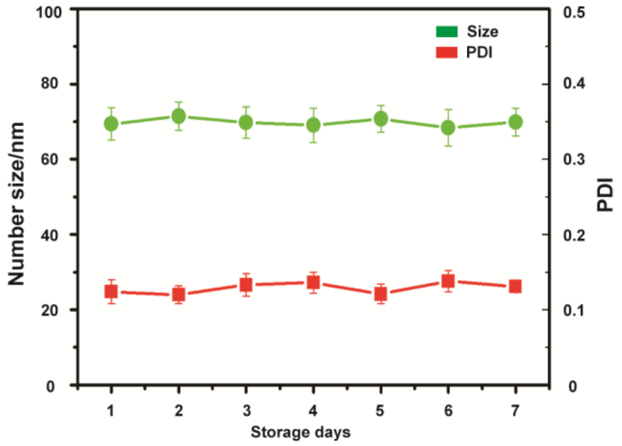

Figure S2. Stability assessment of Pd-MX in distilled water. DLS patterns of Pd-MX in distilled water during storage for 7days.

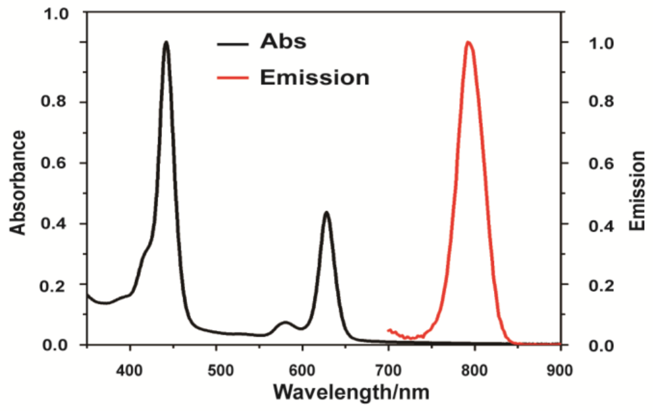

Figure S3. Normalized absorption and phosphorescence emission spectra of Pd-MX (20 μM) in aqueous solution.

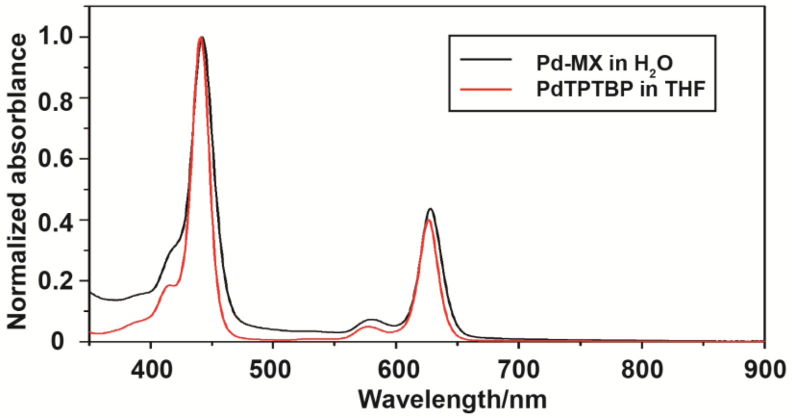

Figure S4. UV-Vis absorption spectrums of Pd-MX in distilled water and PdTPTBP in THF.

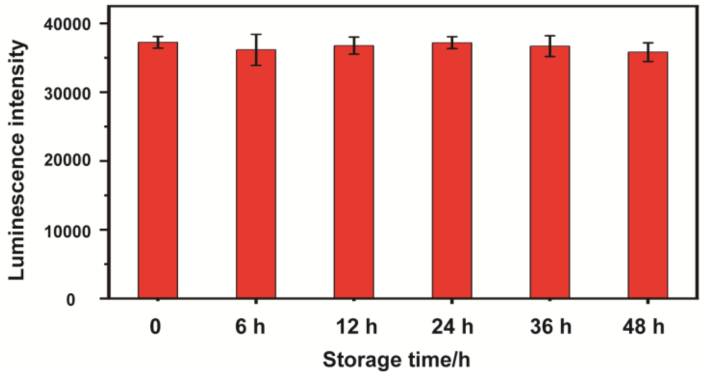

Figure S5. Stability assessment of Pd-MX in 50% mouse serum at 37 ℃. The changes of luminescence intensity of Pd-MX in 50% mouse serum at 37 ℃ during storage for 48 hours.

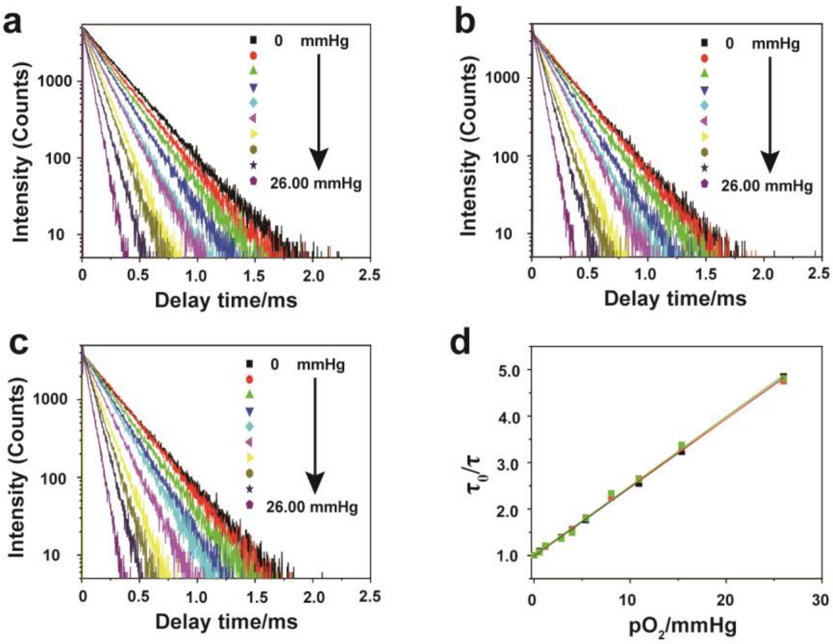

Figure S6. Phosphorescence decays of Pd-MX in aqueous solution (a), in liver tissue homogenate (b) in tumor tissue homogenate (c) at different oxygen levels (0 mmHg, 0.55 mmHg, 1.16 mmHg, 2.82 mmHg, 3.94 mmHg, 5.38 mmHg, 8.04 mmHg, 10.91 mmHg, 15.37 mmHg and 26.00 mmHg) by time-correlated single photon counting (TCSPC) technique in the FLS 920 fluoresence spectrometer. (d) Stern-Volmer plots of τ_0/τ as a function of oxygen pressure in aqueous solution (black), in liver tissue homogenate (red) in tumor tissue homogenate (green). The plots were obtained by time-correlated single photon counting (TCSPC) technique.

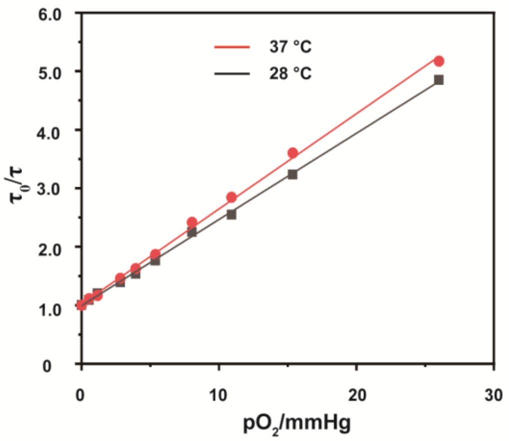

Figure S7. Stern-Volmer plots of τ_0/τ as a function of oxygen pressure (0 mmHg, 0.55 mmHg, 1.16 mmHg, 2.82 mmHg, 3.94 mmHg, 5.38 mmHg, 8.04 mmHg, 10.91 mmHg, 15.37 mmHg and 26.00 mmHg) in 28 °C and 37 °C.

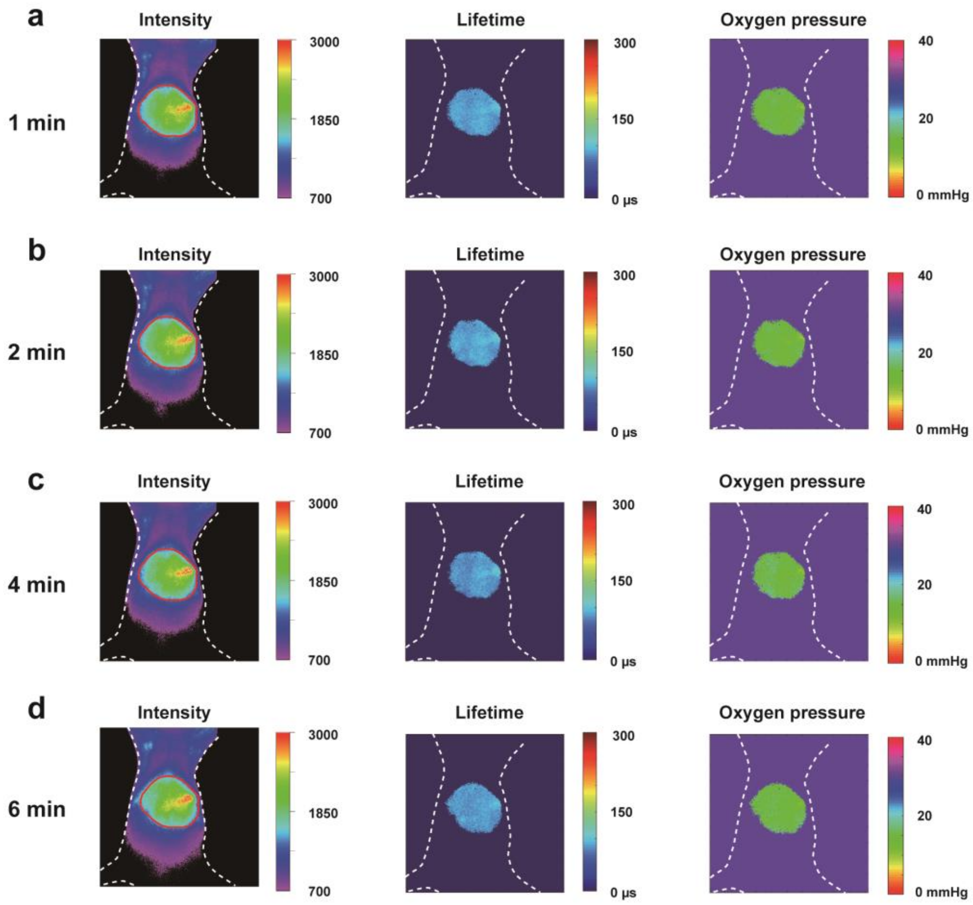

Figure S8. The changes of phosphorescence intensity, phosphorescence lifetime and oxygen pressure in the liver of mouse under 635 nm laser irradiation with different times (a) 1 min, (b) 2 min, (c) 4 min and (d) 6 min after i.v. injection of Pd-MX solution (0.6 mL, 20 μM).

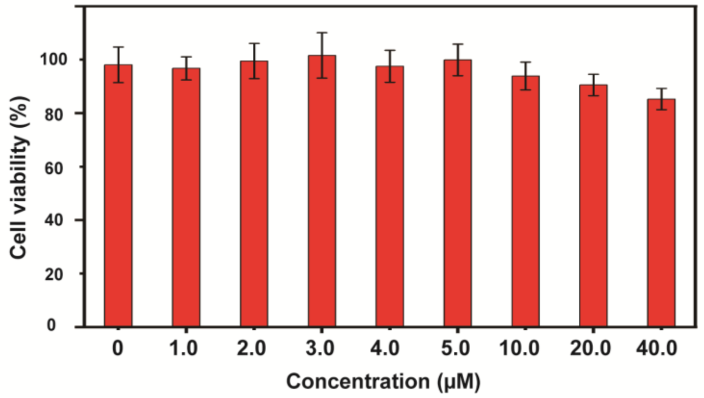

Figure S9. Methyl thiazolyl tetrazolium (MTT) assays of HepG2 cells treated with nanomicelles at different concentrations (0-40 μM). All error bars were defined as s.d. (n = 5).

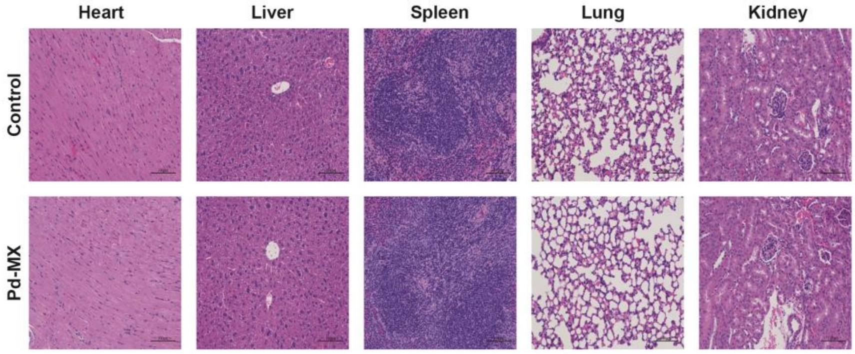

Figure S10. Histological changes in the heart, , liver, spleen, lung, kidney of mice one week after intravenous injection of Pd-MX (0.6 mL, 20 μM). The organs were stained with hematoxylin and eosin (H&E). Scale bars: 100 μm.

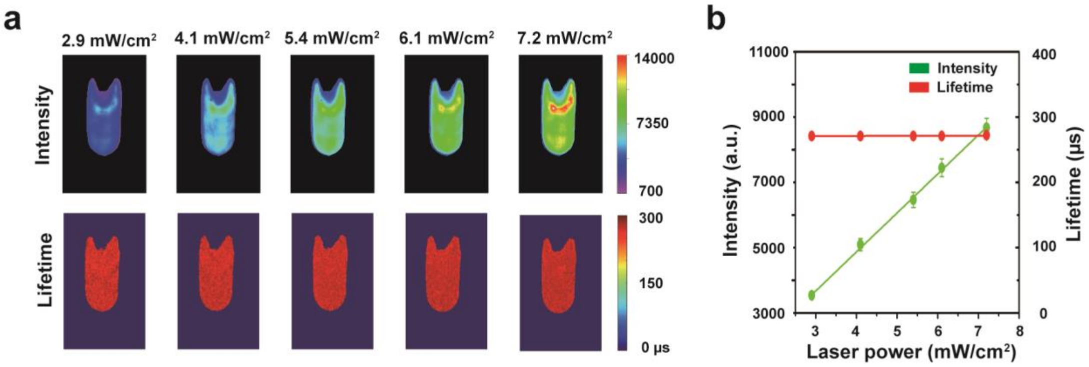

Figure S11. (a) Comparison of phosphorescence intensity imaging (top) and lifetime imaging (bottom) of Pd-MX solution in the vial under 635 nm laser irradiation with different power

density (2.9 mW/cm^2, 4.1 mW/cm^2, 5.4 mW/cm^2, 6.1 mW/cm^2 and 7.2 mW/cm^2). (b) The change tendencies of average phosphorescence intensity and average phosphorescence lifetime of Pd-MX solution in the vial under 635 nm laser irradiation with different power density (2.9 mW/cm^2, 4.1 mW/cm^2, 5.4 mW/cm^2, 6.1 mW/cm^2 and 7.2 mW/cm^2). Each average intensity values or average lifetime values with error bar values was measured at different positions from the corresponding intensity images or lifetime images (n = 5).

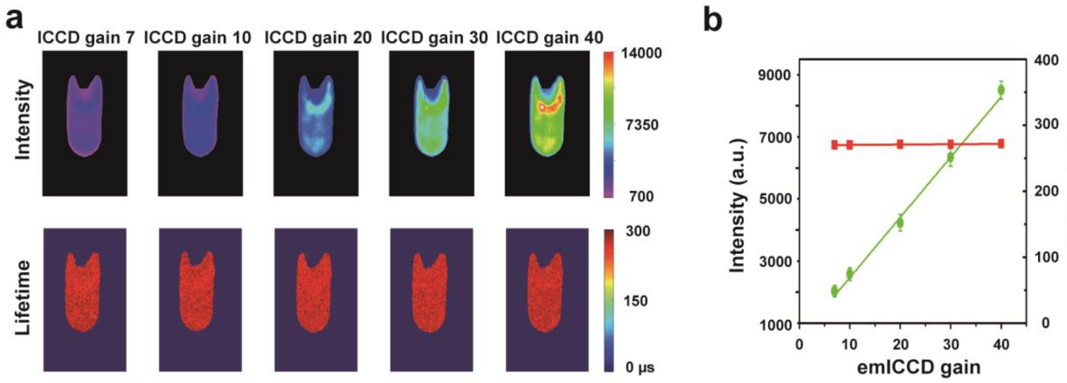

Figure S12. (a) Comparison of phosphorescence intensity imaging (top) and lifetime imaging (bottom) of Pd-MX solution in the vial with different emICCD gain (7, 10, 20, 30 and 40). (b) The change tendencies of average phosphorescence intensity and average phosphorescence lifetime of Pd-MX solution in the vial with different emICCD gain (7, 10, 20, 30 and 40). Each average intensity values or average lifetime values with error bar values was measured at different positions from the corresponding intensity images or lifetime images (n = 5).

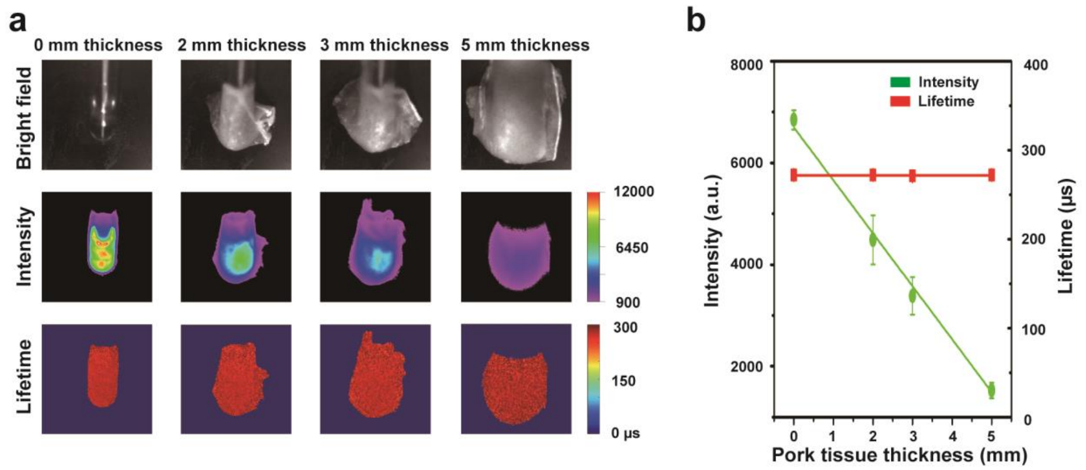

Figure S13. (a) Comparison of phosphorescence intensity imaging (middle) and lifetime imaging (bottom) of Pd-MX solution in the vial under 0 mm, 2 mm, 3 mm and 5 mm thickness pork tissue. (b) The change tendencies of average phosphorescence intensity and average phosphorescence lifetime of Pd-MX solution in the vial under 0 mm, 2 mm, 3 mm and 5 mm thickness pork tissue. Each average intensity values or average lifetime values with error bar values was measured at different positions from the corresponding intensity images or lifetime images (n = 5).

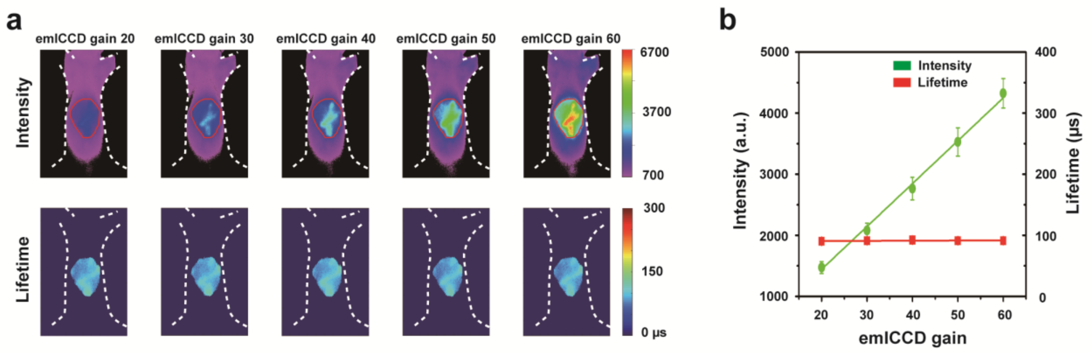

Figure S14. (a) Comparison of phosphorescence intensity imaging (top) and lifetime imaging (bottom) of Pd-MX solution in the liver with different emICCD gain (20, 30, 40, 50 and 60) after i.v. injection of Pd-MX solution (0.6 mL, 20 μM). (b) The change tendencies of average phosphorescence intensity and average phosphorescence lifetime of Pd-MX solution in the

liver with different emICCD gain (20, 30, 40, 50 and 60) after i.v. injection of Pd-MX solution (0.6 mL, 20 μM). Each average intensity values or average lifetime values with error bar values was measured at different positions from the corresponding intensity images or lifetime images (n = 5).

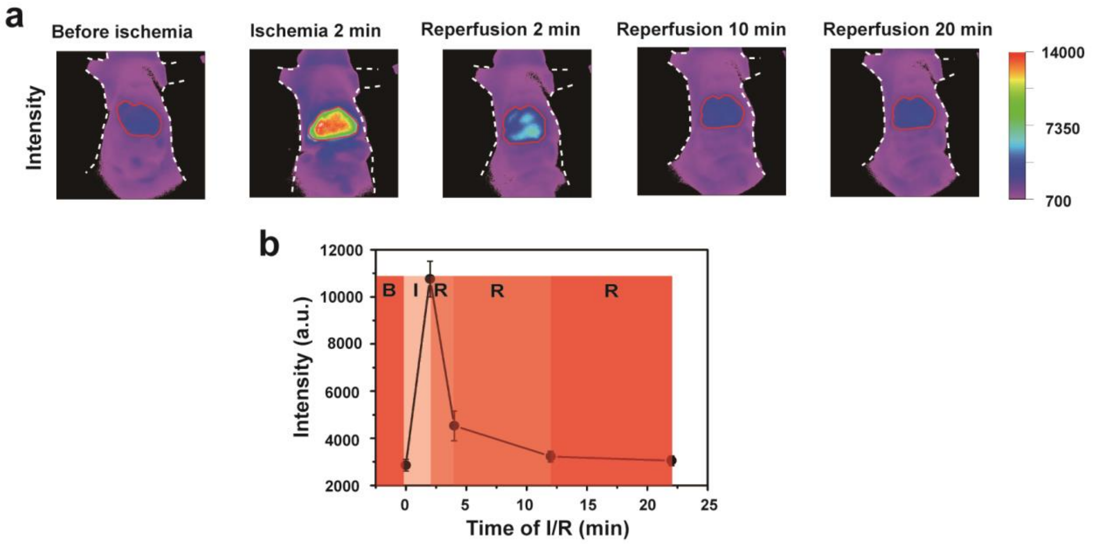

Figure S15. (a) Phosphorescence intensity imaging of mouse liver recorded during ischemia-reperfusion (before ischemia, ischemia 2 min, reperfusion 2 min, reperfusion 10 min and reperfusion 20 min). (b) The change tendency of average phosphorescence intensity of mouse liver obtained before ischemia, ischemia 2 min, reperfusion 2 min, reperfusion 10 min and reperfusion 20 min. Each average intensity values with error bar values was measured at different positions from the corresponding intensity images (n = 5).

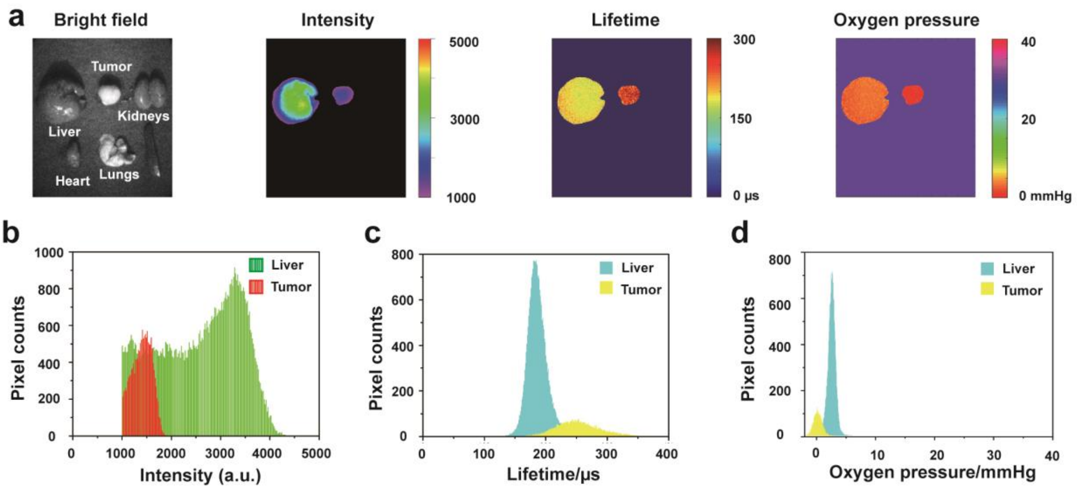

Figure S16. (a) Imaging of isolated organs from the executed mouse bearing subcutaneous tumor, including heart, liver, spleen, lungs, kidneys and tumor. (b) Phosphorescence intensity histograms of the isolated tumor region and isolated liver region from the executed mouse bearing subcutaneous tumor. (c) Phosphorescence lifetime histograms of the isolated tumor region and isolated liver region from the executed mouse bearing subcutaneous tumor. (d) The oxygen presure histograms of the isolated tumor region and isolated liver region from the executed mouse bearing subcutaneous tumor.

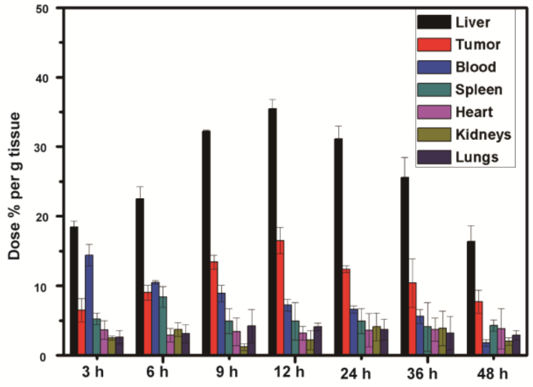

Figure S17. Biodistribution of Pd-MX in different organs at various time points after i.v. injection of the probe. The values were presented as the percentage of injected dose per g of collected organ and based on three mice per group.

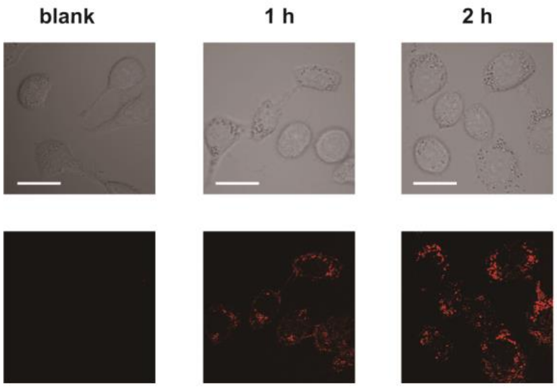

Figure S18. Fluorescence imaging in HepG2 cells using nanoprobe Pd-MX. (left) HepG2 cells only; (middle) HepG2 cells incubated with Pd-MX (5 μM, 1 h); (right) HepG2 cells incubated with Pd-MX (5 μM, 2 h).

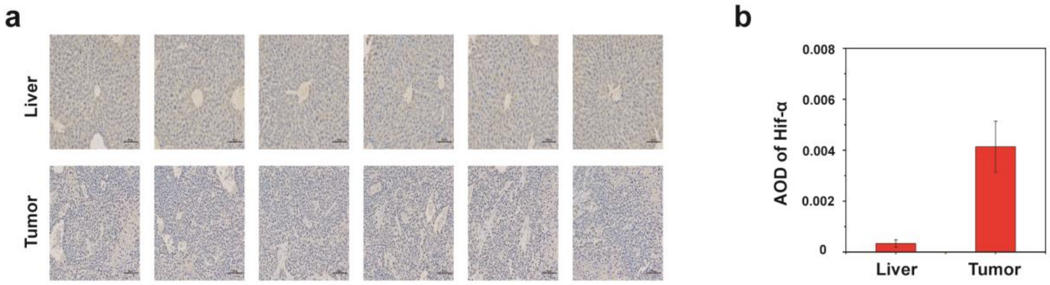

Figure S19. (a) IHC staining of HIF-1α in the regions of of liver and in the regions of tumor from the executed mouse bearing subcutaneous tumor. Scale bar: 100 μm. (b) Analysis of the IHC staining with hypoxia-inducible factors (HIF-1α) antibody by comparison of average optical density (AOD) between the regions of liver and the regions of tumor from the executed mouse bearing subcutaneous tumor.

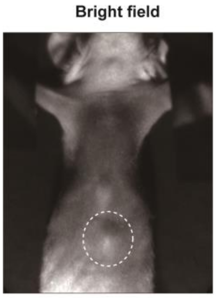

Figure S20. Bright field imaging of the living mouse bearing orthotopic tumor. White cycle, a region of interest (ROIs) that was performed for ultrasound imaging.

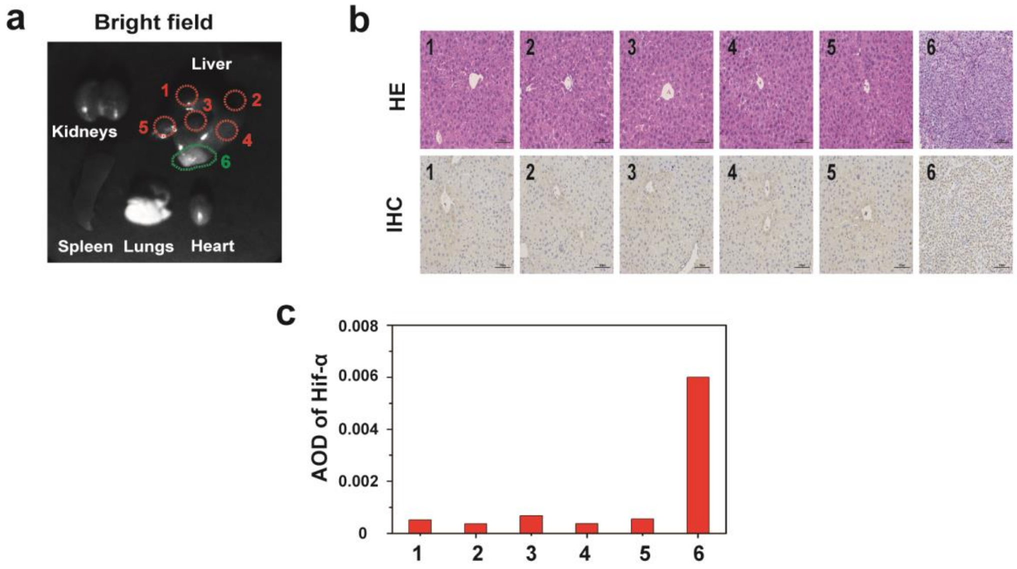

Figure S21. (a) Imaging of isolated organs from the executed mouse bearing orthotopic liver tumor, including heart, spleen, lungs, kidneys and liver. Red cycles (Nos.1-5), regions of interest (ROIs) of normal liver. Green cycle (Nos.6), region of interest (ROIs) of tumorous liver. (b) HE staining and IHC staining of HIF-1α in the regions of interest (ROIs) of normal liver (Nos.1-5) and in the region of interest (ROIs) of tumorous liver (Nos.6) from the executed mouse bearing orthotopic liver tumor. Scale bar: 100μm. **(c)** Analysis of the IHC staining with hypoxia-inducible factors (HIF-1α) antibody by comparison of average optical density (AOD) between the regions of interest (ROIs) of normal liver (Nos.1-5) and the region of interest (ROIs) of tumorous liver (Nos.6) from the executed mouse bearing orthotopic liver tumor.

Mouse		Average lifetime	Average oxygen pressure
Mouse 1	Before ischemia	84 μs	15.0 mmHg
	2 min ischemia	171 μs	4.0 mmHg
	2 min reperfusion	106 μs	10.6 mmHg
	10 min reperfusion	84 μs	15.0 mmHg
Mouse 2	Before ischemia	95 μs	12.5 mmHg
	2 min ischemia	192 μs	2.9 mmHg
	2 min reperfusion	106 μs	10.6 mmHg
	10 min reperfusion	98 μs	12.0 mmHg
Mouse 3	Before ischemia	104 μs	10.9 mmHg
	2 min ischemia	172 μs	4.0 mmHg
	2 min reperfusion	121 μs	8.4 mmHg
	10 min reperfusion	105 μs	10.7 mmHg

Table S1 Statistics assessment for the hepatic ischemia-reperfusion model in living mice (n = 3).

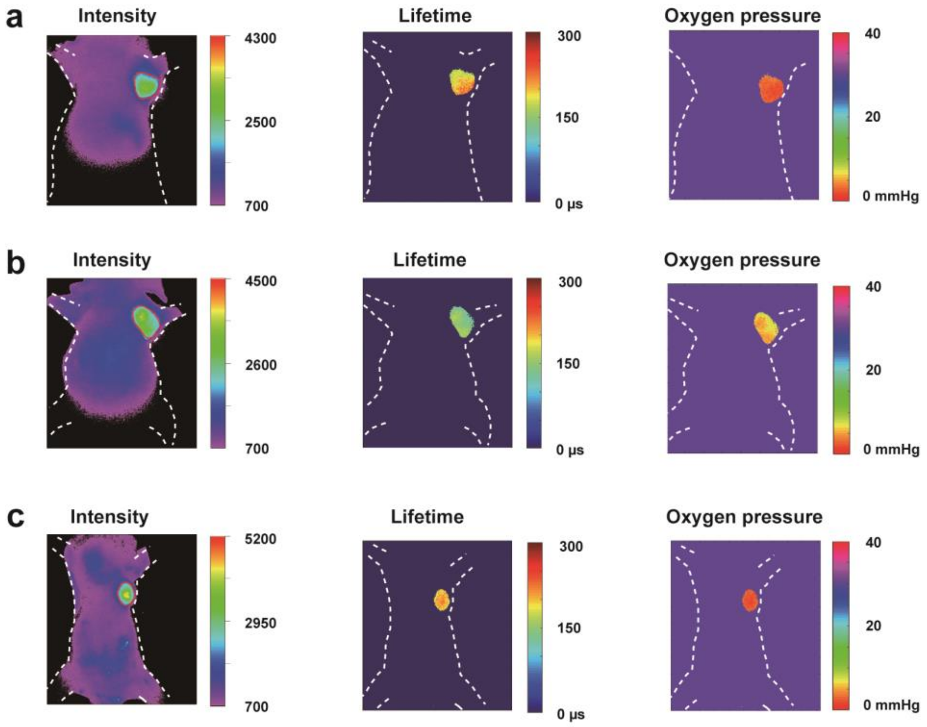

Figure S22. Imaging of the living mice bearing subcutaneously implanted HepG2 tumors with three sizes after 12 h i.v. injection of Pd-MX (0.6 mL, 20 μM), (a) tumor size: 0.35 g; tumor size: 0.56 g; tumor size: 0.22 g.

Mouse		Average lifetime	Average oxygen pressure
Mouse 1	tumor	192 μs	2.9 mmHg
	liver	98 μs	12.0 mmHg
Mouse 2	tumor	141 μs	6.3 mmHg
	liver	101 μs	11.4 mmHg
Mouse 3	tumor	202 μs	2.4 mmHg
	liver	104 μs	10.9 mmHg

Table S2 Statistics assessment in the living mice bearing subcutaneously implanted HepG2 tumors (n =3).

Mouse		Average lifetime	Average oxygen pressure
Mouse 1	tumorous liver	150 μs	5.5 mmHg
	normal liver	96 μs	12.4 mmHg
Mouse 2	tumorous liver	155 μs	5.1 mmHg
	normal liver	94 μs	12.7 mmHg
Mouse 3	tumorous liver	172 μs	4.0 mmHg
	normal liver	110 μs	10.0 mmHg

Table S3 Statistics assessment in the living mice bearing orthotopic liver tumor (n = 3).

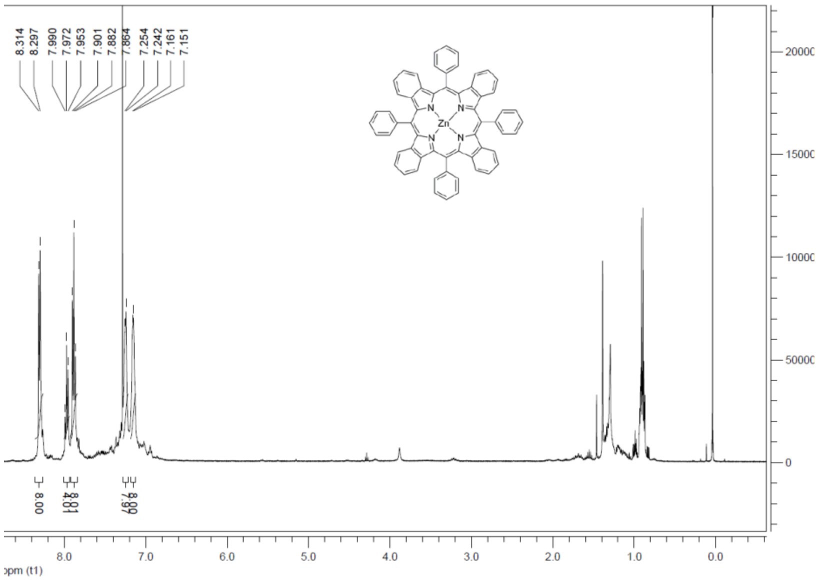

Figure S23. ^{1}H NMR chart of the compound of ZnTPTBP ($CDCl_3$, 400 MHz).

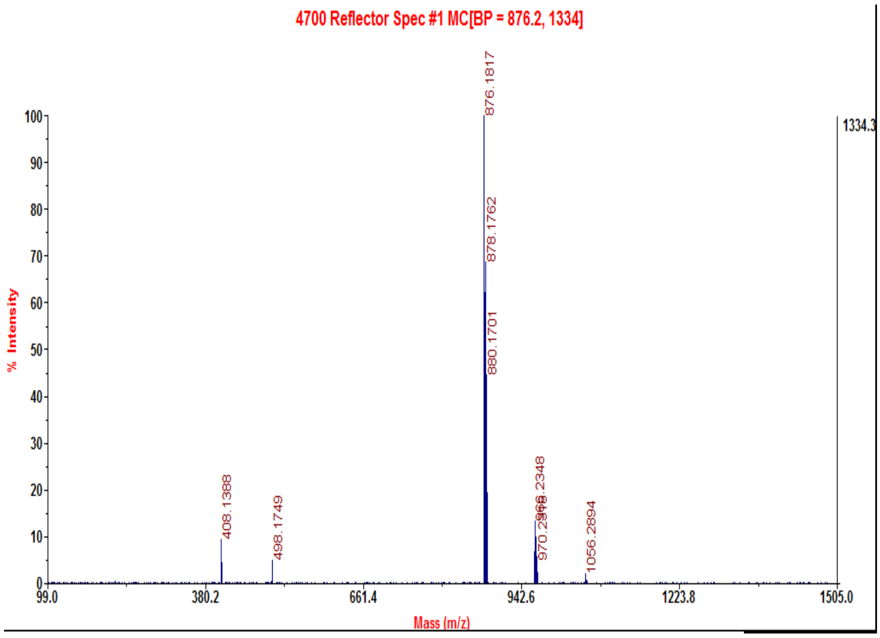

Figure S24. Maldi-Tof/Tof-MS spectrum of the compound ZnTPTBP.

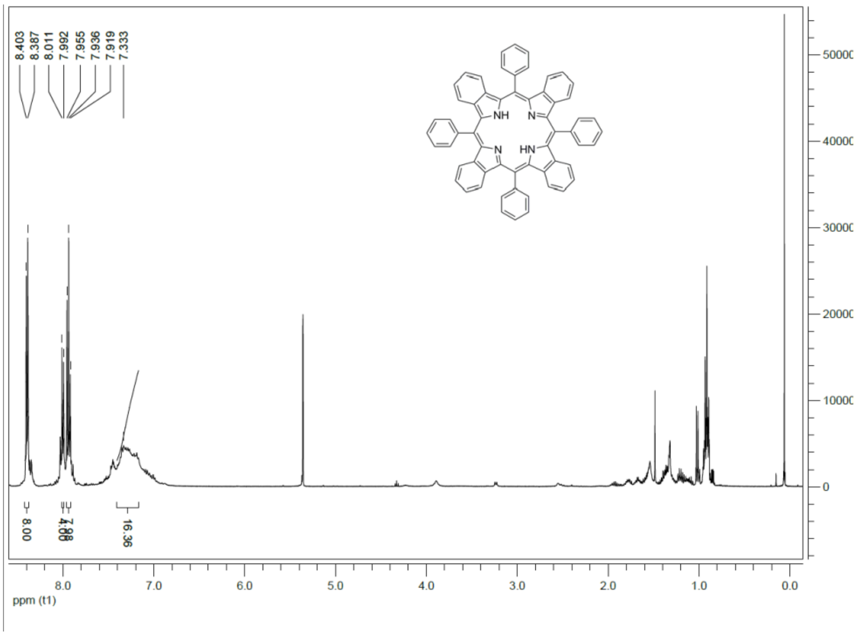

Figure S25. ^{1}H NMR chart of the compound of H_2TPTBP (CD_2Cl_2, 400 MHz).

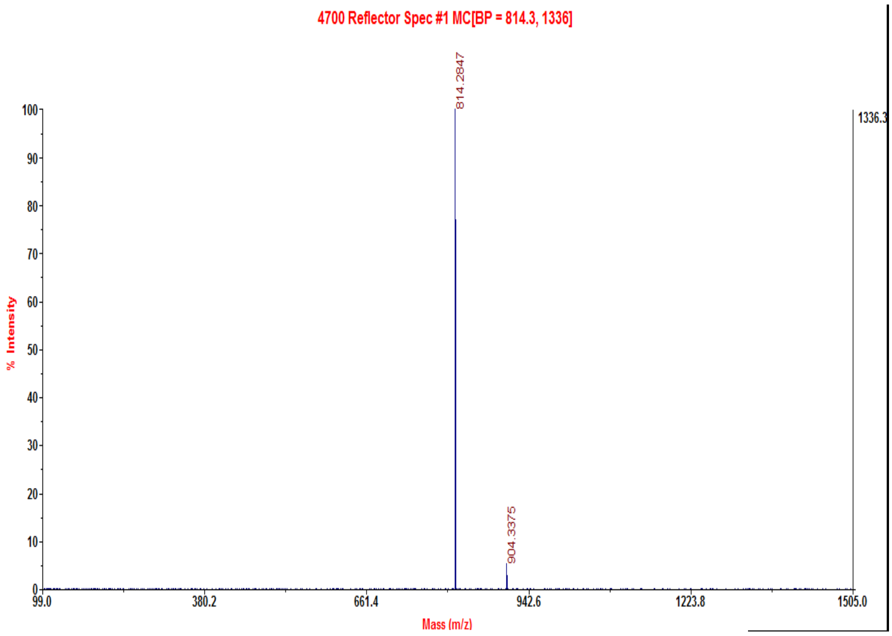

Figure S26. Maldi-Tof/Tof-MS spectrum of the compound H_2TPTBP.

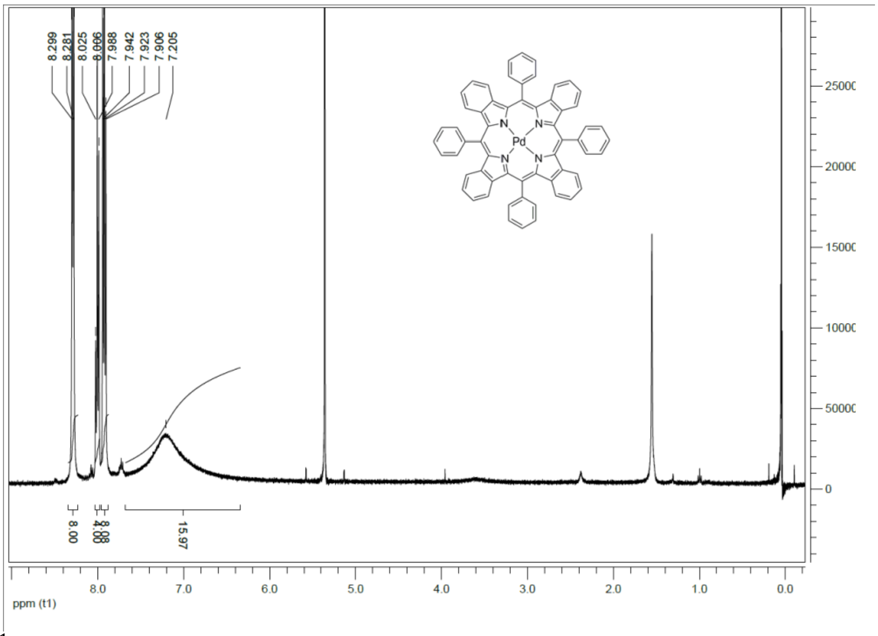

Figure S27. ^{1}H NMR chart of the compound of PdTPTBP (CD_2Cl_2, 400 MHz).

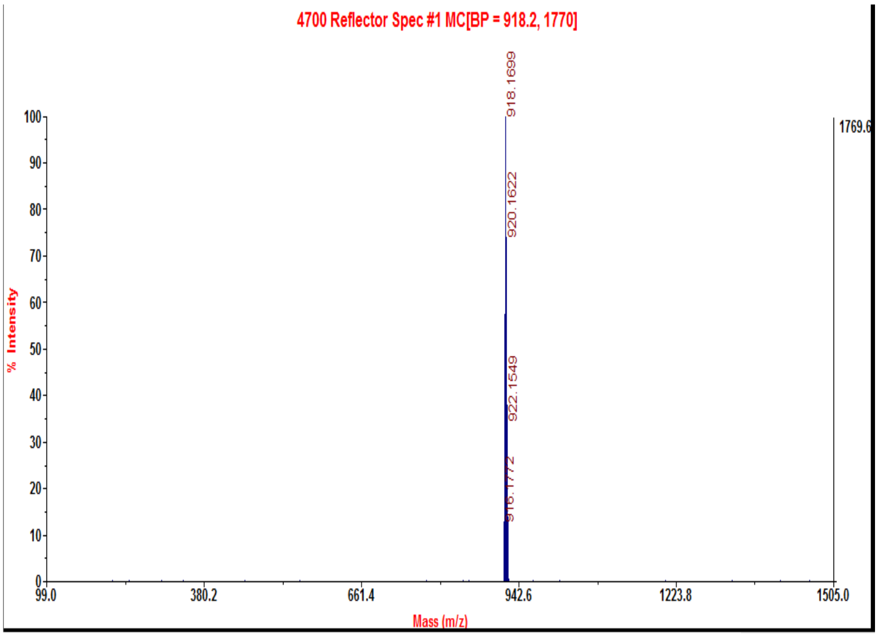

Figure S28. Maldi-Tof/Tof-MS spectrum of the compound PdTPTBP.

References

1. S. M. Borisov, G. Nuss, W. Haas, R. Saf, M. Schmuck, I. Klimant, *J. Photochem. Photobiol., A*, **2009**, *201*, 128.

2. X. Cui, J. Zhang, P. Yang, J. Sun, *Chem. Commun.*, **2013**, *49*, 10221.
